# A Review of Patents and Innovative Biopolymer-Based Hydrogels

**DOI:** 10.3390/gels9070556

**Published:** 2023-07-07

**Authors:** Snežana Ilić-Stojanović, Ljubiša Nikolić, Suzana Cakić

**Affiliations:** Faculty of Technology, University of Niš, Bulevar Oslobodjenja 124, 16000 Leskovac, Serbia; nljubisa@tf.ni.ac.rs (L.N.); cakics@tf.ni.ac.rs (S.C.)

**Keywords:** fibrin, silk fibroin, collagen, keratin, gelatin, chitosan, hyaluronic acid, alginate, carrageenan, cellulose

## Abstract

Biopolymers represent a great resource for the development and utilization of new functional materials due to their particular advantages such as biocompatibility, biodegradability and non-toxicity. “Intelligent gels” sensitive to different stimuli (temperature, pH, ionic strength) have different applications in many industries (e.g., pharmacy, biomedicine, food). This review summarizes the research efforts presented in the patent and non-patent literature. A discussion was conducted regarding biopolymer-based hydrogels such as natural proteins (i.e., fibrin, silk fibroin, collagen, keratin, gelatin) and polysaccharides (i.e., chitosan, hyaluronic acid, cellulose, carrageenan, alginate). In this analysis, the latest advances in the modification and characterization of advanced biopolymeric formulations and their state-of-the-art administration in drug delivery, wound healing, tissue engineering and regenerative medicine were addressed.

## 1. Introduction

Nowadays, there is great research motivation regarding materials based on biopolymers due to the desire to replace the use of traditional polymers and monomers originated from petroleum, the resources of which are limited. The other problem with synthetic polymers is the removal of accumulated waste plastics and their hazardous and toxic residues after decomposition in the environment [1]. Additionally, excessive petroleum applications cause the adoption of strict ecological laws for environmental safety [2]. Unlike synthetic polymers, biopolymers have numerous benefits, e.g., they usually degrade into non-dangerous substances in the environment. There is a focus in contemporary scientific studies on the development of natural polymer materials that are biodegradable, safe, biocompatible and available from renewable and sustainable resources. Biomaterials present a class of materials made to naturally interact with body fluids, living tissues and supporting cells [3]. They have been used as therapeutics in numerous fields of medicine (e.g., in surgery, orthopedics, dentistry) with the intention to heal, restore damaged or replace lost human body functions (such as drug delivery systems, implants, devices or prostheses) or in diagnostics. The term biopolymer appertains to all polymers synthesized by living organisms (microorganisms, algae, plants, animals). Originating inside living organisms, they are composed of numerous covalently linked repeating units, monomers, and build cells and tissue structures that can grow, propagate and regenerate. Biopolymers have a number of functions in living organisms. Certain biopolymers build connective tissue; help in the tissue function, e.g., human cartilage; while others provide molecules applied as signals to trigger the endocrine system [4]. Due to their natural origin and superior biochemical, mechanical and thermal properties, biopolymers are suitable in numerous pharmaceutical and biomedical applications. The extracellular matrix, as the natural surroundings of the cells, is a type of native biopolymer hydrogel.

By definition, hydrogels are known as three-dimensional insoluble supramolecular or covalent polymer networks that can hold a great quantity of water or fluids due to the balance in the osmotic pressure forces and the elastic forces of the crosslinked macromolecular chains. Usually, they are formed by physical or covalent interactions of hydrophilic macromolecules. Biopolymer-based hydrogels can be built from biopolymers soluble in physiological fluids, involving natural proteins (e.g., fibrin, silk fibroin, collagen, keratin, gelatin,) and natural polysaccharides (e.g., hyaluronic acid, cellulose, carrageenan, alginate, pectin, chitosan) by the crosslinking process [5]. Hydrogels can be crosslinked by physical, covalent or ionic bonds. The chemical composition and density of the hydrogels’ network affect the swelling process, release velocity and kinetics of the absorbed fluid and active ingredient. Voluminous functional groups are particularly important because they lead to intramolecular and intermolecular physical interactions (hydrogen bonds, hydrophobic interactions, dipole–dipole interactions). Stimuli-sensitive hydrogels are called “smart hydrogels” and manifest a notable transition in their characteristics due to small changes in their outside surroundings (temperature, light wavelength, pH value, ultrasound, ionic strength, magnetic or electric fields as well as their combinations) [6]. Their reactions to outside stimuli present as a change in at least one feature, e.g., degree of swelling, morphology characteristics, mechanical properties, volume, shape, network structure, degradation, permeability or phase transitions. The unique hydrogel properties of elasticity and flexibility, bioadhesion, superior biocompatibility, swelling properties, specific response to stimuli and soft structure are similar to the structures of living tissues [7]. The temperature sensitivity of hydrogel biomaterials is the most studied aspect for controlled drug release. A special feature of thermosensitive hydrogels is their critical solution temperature. Negative thermosensitive hydrogels have a lower critical solution temperature (LCST) as the critical temperature under which the hydrogel swells in fluid. Above a lower critical solution temperature, the hydrogel contracts and becomes progressively hydrophobic, leading to hydrogel transition. Positive thermosensitive hydrogel has an upper/higher critical solution temperature (UCST/HCST). Positive thermosensitive hydrogel contracts upon cooling under the higher critical solution temperature. The pH-sensitive hydrogels have ionizable lateral functional groups with the ability to relieve or receive protons depending on surrounding pH value changes. A small change in fluid ionic strength and/or pH value can trigger a significant change in their properties, e.g., their degree of swelling. Cationic hydrogels generally contain lateral amino groups, swell below pKb values and contract above pKb values. Anionic hydrogels generally have carboxylic or sulfonic lateral groups and swell in the fluid at the pH value which is above the pKa values. The amphiphilic hydrogels contain anionic and cationic lateral groups [6]. Dual thermo- and pH-responsive hydrogels carry great importance because temperature and pH value are the parameters that change in a living body most often. Hydrogels have significant practical applications in pharmaceuticals, food and agriculture industries, biomedicine and biosensors, and this study presents only part of the trends in the current research.

Biopolymer-based hydrogels are applied in drug delivery systems as formulations or carriers that allow for the encapsulation of therapeutic active substances and the control of transport through biological membranes to the site of action for the treatment of diseases with improved efficiency and safety [7]. They can incorporate drugs when they swell and release them in the contracted state, e.g., at higher temperatures (LCST hydrogels) or at a low pH value, i.e., in the stomach (cationic hydrogels) [6]. Additionally, they are used in the field of tissue engineering in the form of a matrix capable of sustaining the cell’s life (undifferentiated and differentiated) in its three-dimensional structure. Biopolymer-based hydrogels have various uses, e.g., as cell culture substrates, in regenerative medicine, as wound dressing, as an implant and in pharmaceutical or cosmetic compositions [6]. Generally, degradation products of natural polymers (proteins, nucleic acid and polysaccharides) are not harmful. Contemporary research and development are focused on the investigation of self-healing hydrogels, an application for bio-inks invented for 3D bioprinting [8]. Scientists are trying to design hydrogels in order to additionally mimic living systems for advanced applications in medicine. Nowadays, investigations into hydrogels represent scientists’ efforts to invent a new, innovative and applicable hydrogel structures and properties for use in sophisticated materials [9,10].

This review aims to present the latest achievements in this field, comprising an analysis of patent documents and published scientific works in the field of biopolymer-based hydrogels. In addition to information contained in scientific journals, the information contained in patent documents is also very interesting as a source of significant technical information. The technical knowledge which can be found there has not yet been published anywhere else. Patent documentation databases can serve as both problem-solving resources and inspiration for future studies. After the examination procedure, a patent is granted for an invention that is new (an identical solution was not available to the public before the date of filing the patent application), includes an inventive step (it is not obvious compared to known solutions in the relevant technical field) and finally, is industrially applicable (it can be produced or industrially used). A patent gives its holder the exclusive right to use the protected invention in the territory of the country that recognized it, as well as the right to prevent others from using that invention for commercial purposes [11,12]. The criteria for choosing the analyzed patent documents in this review included the newest-granted patents and the patenting levels. This review highlights the newest innovative inventions, their most important features, as well as recent developments and recommendations that can help in planning an innovative research strategy.

**Figure 1:**
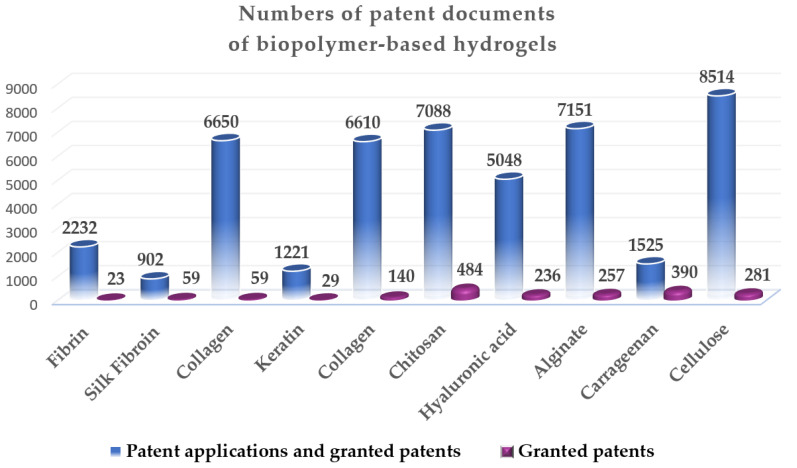
Numbers of patent documents (patent applications, granted patents) of biopolymer-based hydrogels from 1915 to May 2023. Data were obtained using the Espacenet database [13].

A review was carried out using worldwide Espacenet [13] and Scopus [14] databases (accessed in May 2023) to access the selected patent documents and recent articles for bibliometric analysis. In this review, the Advanced search and Filters toggle options on the new Espacenet advanced search service of the European Patent Office (EPO) were applied [13]. The methodological procedure for the bibliometric evaluation was divided into two main phases: the data collection phase, and the data mapping/visualization phase. Throughout this research, defined terms and keywords (hydrogel, biopolymer, polysaccharide, protein, fibrin, silk fibroin, collagen, keratin, gelatin, chitosan, hyaluronic acid, alginate, carrageenan, cellulose) were used. Patent documents were researched by title, abstract and claims. The entered query string included English, German and French languages; the terms in field title, abstracts and claims were combined (e.g., cellulose hydrogel or cellulose biopolymer) as search words. In order to refine this search, some areas were excluded (e.g., accounting, mathematics, economics, finance, management, business, energy, computer science, social science, textile, neuroscience, planetary science, nursing, and health professions) as well as certain words (e.g., oleogels, aerogels, female, male). During the patent data search, 46,941 patent documents were found, comprising 44,983 patent applications and 1958 granted patents for biopolymer-based hydrogels (from 1915 to May 2023). After that, the research was narrowed to involve only granted patents (includes as a filter “B” for publication number) with keywords in the title and with publication dates after 2010, and the documents were sorted by relevance. Subsequently, the search was conducted by combining different keywords for application (e.g., drug delivery, wound healing, tissue engineering, regenerative medicine, food) to further selection. Finally, the types of documents were considered: registered patents (in the Espacenet database), articles and reviews (in Scopus). The search was further filtered (if it was necessary) to involve only articles and reviews with publication dates after 2020. Following this research, the numbers of patent documents according to the type of biopolymer-based hydrogels analyzed in this review are presented in Figure 1.

## 2. Hydrogels Based on Natural Polymers

### 2.1. Natural Proteins

Proteins are compounds of high molecular weight consisting of amino acids interconnected by peptide bonds. They are the main structural components of the human organism [15]. Polypeptides, usually called proteins, are built of numerous amino acids that are covalently linked over amide bonds. Based on their amino acid chain conformation, protein structures are divided into four levels: primary, secondary, tertiary or quaternary. The characteristics unique to each protein are the composition of amino acid, their size, subunit structures, sequence, shape, solubility, net charge, heat stability, hydrophobicity and isoelectric point. Depending on these properties, different methods of isolation and purification were developed which are important for their use. Natural proteins are important as biopolymer-based hydrogel materials because of their unique properties. This review analyzes fibrin, silk fibroin, collagen, keratin and gelatin.

#### 2.1.1. Fibrin

Fibrin is an insoluble, non-globular protein in the form of long fibrous chains, formed in the process of soluble protein fibrinogen conversion via protease thrombin enzymes in the process of blood coagulation. Fibrin was discovered by Marcello Malpighi in 1666 [16]. The first enzymatic stage is characterized by the thrombin-catalyzed scission of fibrinopeptides from fibrinogen to form a monomer called fibrin. This process causes the monomeric fibrin molecules to self-assemble spontaneous polymerization at the non-enzymatic step. They form fibrin oligomers which elongate to build protofibrils. These protofibrils are two-stranded and aggregate longitudinally and laterally to establish branched fibers and building up a sponge-like, gelled, three-dimensional interconnected structure that entangle platelets. This mass gradually hardens and contracts to form a blood clot over a wound site, which is essential for hemostasis [17]. Eventually, the fibrin polymer is crosslinked over covalent bonds, stabilized by plasma transglutaminase (Factor XIIIa), and it then forms a mature fibrin clot which is much more stable, both chemically and mechanically. Because of its fast crosslinking and glue-like gel form, fibrin has been largely applied in many areas, i.e., as a surgical glue, sealant and hemostatic agent [18]. The hydrogels based on fibrin (fibrinogen) were applied in scaffolds, tissue culture, promotion bone growth and healing and regenerative medicine [19]. The available documents highlight its good biodegradability, biocompatibility and weak but tunable mechanical and nanofibrous structural characteristics. Fibrin is a preferable material for bio-inks, and due to its non-linear elasticity, it easily allows for intercellular communication [8]. It stimulates cell migration, osteoconduction and vascularization. In vitro degradation rates were reduced by fibrinolytic inhibitors (i.e., aprotinin or aminocaproic acid). Fibrin is applied in cardiac tissue engineering, skin regeneration and growth factor incorporation [5].

In the worldwide Espacenet database [13] for fibrin biopolymer hydrogels, based on the defined criteria, 2232 patent documents were found (granted patents and patent applications), filtered according to title, abstract and claims from 1915 to 27 May 2023. Obtained data were additionally filtered in order to include only documents with the keyword “fibrin” in the title and an earliest priority date after 2013. As a result, 23 granted patents were found. Some of the documents selected according to their relevance are summarized in Table 1 and further analyzed.

The novelty of registered patent ES2527800B1 is its photothermal composition suitable for generating hyperthermia in biological tissues in which this composition is implanted [20]. This patent relates to the application of this composition for tumor destruction, infection treatment or tissue regeneration, as well as for the controlled delivery of therapeutic agents. The present invention relates to a composition comprising a fibrin hydrogel matrix or a mixture of its precursors, thrombin and fibrinogen, in which plasmid nanoparticles and thermosensitive effectors are embedded, which contain therapeutic agents which are released after application. The plasmon nanoparticle is distributed in the fibrin matrix homogeneously. Effectors consist of liposomes, genetically modified cells (and any combination thereof) and therapeutic agents that are released by applying electromagnetic radiation.

The subject of registered patent US11371021B2 is the production of tissues and cell cultures from stem cells (pluripotent and undifferentiated) by PEG-fibrinogen hydrogels as three-dimensional biomimetic materials [21]. The microenvironment is three-dimensional, and it possesses a diversity of structures or shapes together with microislands, strings, microspheres, cardiac discs and macrotissues. The compositions and novel methods ensure novel cell delivery platforms and viable cell sources which allows for the substitution of diseased tissue and new cardiomyocytes engraftment from sources available in vitro. The three-dimensional, synchronously contracting cardiac tissue, as a single unit, contracts spontaneously with a contraction frequency band from 0.59 to 1.53 Hertz.

Patent CN106581772B protects a cartilage repair material—fibrin/hyaluronic acid hydrogel, which contains granulocyte colony-stimulating factor (gelatin microspheres) and pharmaceutically acceptable auxiliary materials or components [22]. The invention also discloses their preparation method and a new application of the granulocyte colony-stimulating factor.

Registered patent KR101991035B1 relates to an optimized three-dimensional network of fibrin–poloxamer polymer composite hydrogel and a method for its preparation and application in a scaffold for tissue regeneration [23].

The fibrin biopolymer-obtaining process, protected by patent BR102017008027B, was described in 13 steps, and was characterized by providing the dehydration of serine protease (purified from snake venom or the synthesized equivalent) and fibrinogen-rich cryoprecipitate (extracted from large animals) obtained using drying, filtration, mechanical pressing, osmotic dehydration, lyophilization or similar [24].

Patent CN110947034B relates to a bioactive calcium phosphate–fibrin composite hydrogel for injectable bone repair, which includes a mixed gelling system based on bioactive calcium phosphate, fibrin and thrombin [25]. Biologically active calcium phosphate comprises amorphous phosphate base and phosphorus-containing base biomolecules and/or hydrolyzates of phosphorus-containing base biomolecules, which are uniformly complexed with the amorphous calcium phosphate.

Fibrin has been largely applied as a biopolymer hydrogel, alone or in combination with other materials. Due to its advantages and unique physical and biological characteristics (biodegradability, biocompatibility, porosity, elasticity), it has usually been applied as a scaffold (for cells to regenerate tissue, bone, cardiac tissue, cartilage, skin), surgical glue, sealant and hemostatic agent for vascularization and osteoconduction. Its main weaknesses are its mechanical and nanofibrous structural characteristics and fast biodegradability. Additionally, fibrinogen has poor printable properties, e.g., high viscosity of crosslinked fibrin, which hinders proper ink extrusion and is not capable of maintaining the 3D shape of bio-printed constructs. With the goal of overcoming these weaknesses of fibrin-based biopolymers for biomimetics and regenerative usage, different strategies were applied, generally in various compositions, with several natural or synthetic polymers. Hydrogels based on fibrin, alone or as biopolymer composites in combination with calcium phosphate, Poloxamer, hyaluronic acid, plasmonic nanoparticles, polyetilenglycol, photothermal effectors, or bioactive calcium phosphate, are described in selected patents. They have been used in composition for tumor destruction, infection treatment or tissue regeneration, for the controlled delivery of therapeutic agents, the substitution of diseased tissue and cardiomyocytes engraftment, as cartilage repair material, as a scaffold for tissue regeneration, for injectable bone repair and for surgical cells implantation. New investigations and strategies are required in order to improve the physical and chemical features of fibrin-based hydrogels in the future.

#### 2.1.2. Silk Fibroin

Silk (silk fibroin) is an insoluble, natural, fibrous protein, derived by certain insects and arachnids as a building material for cocoons and webs. Hierarchical self-organized silk chains are comprised of alternating hydrophobic and hydrophilic regions like a block polymer, which provides amphiphilic properties and the ability to build semicrystalline structures by crosslinking and hydrophobic interactions. Silk from various natural sources has been used as a biomaterial without any additional changes. For commercial and biological use, silk is almost fully restricted to filaments from the cocoons of domesticated silkworms (caterpillars of several moth species from the genus *Bombyx*) [26,27]. Silk fibroin, a unique protein, is applied as a prospective biopolymer due to its excellent biocompatibility and degradability. It is harmless, non-toxic and without immune response. Silk fibroin is stable at physiological conditions (temperatures and pH values). It is insoluble in the majority of solvents (organic and aqueous). After film formation, the texture of pure silk fibroin is fragile, but its mechanical properties are similar to, or usually better than, numerous high-performance synthetic fibers. Fibroin is secreted by the two silk glands of silkworms. Hydrogels based on silk are promising biomaterials, especially for growing tissue grafts for application in regenerative medicine and tissue engineering [5,8,28,29]. Silk scaffolds are used in bone regeneration [30] and 3D bioprinting [31]. Silk is also applied in controlled drug release [32] and wound healing [33].

Silk fibroin, as a natural biopolymer hydrogel, was the subject of 902 patent documents (patent applications and granted patents) from 1981 to May 2023 in the worldwide Espacenet database according to title, abstract and claims. In order to narrow this search, abstract and claims were excluded, as well as documents with a publication date prior to 2013. A total of 96 patent applications were found (since 1988), from which 59 patents have been granted. In the last ten years (from 2013 to 2023), 40 patents were granted, and selected relevant documents are described and summarized in Table 2.

Patent KR101462485B1 relates to a hydrogel composition for treating skin burns. It is prepared using silk fibroin extracted from silkworm cocoons, sodium carboxymethyl cellulose as a first gelling agent and alginate metal salt (sodium alginate, potassium alginate) or calcium alginate as a second gelling agent [34]. This hydrogel composition is excellent for skin burn treatments as it causes pain relief, the prevention of heat and moisture loss and the prevention of secondary infection and, in particular, the prevention of scars.

A silky hydrogel mask is a subject of the TWI609699B patent, and it is obtained via the following steps: dissolving silk fibroin powder in an aqueous urea solution, gelling the formed composition and forming the gelled product into a desired shape [35].

The carbonic anhydrase-fixed silk hydrogel, or a composition including it, according to patent KR101745369B1, is double crosslinked by photocrosslinking and alcohol treatment; hence, it is both eco-friendly and economical, has an excellent thermal and storage stability and can be used repeatedly [36]. The enzyme activity is excellent, so it has the effect of removing, converting, or fixing carbon dioxide.

The photocrosslinked silk fibroin composite hydrogel, prepared by patent CN106977670B, has wide application possibilities in biomedicine, tissue engineering and similar [37].

Patent CN107619481B discloses an interpenetrating network silk fibroin hydrogel and a preparation method thereof [38]. Horseradish peroxidase catalyzes the polymerization reaction of N-vinylpyrrolidone monomer to generate polyvinylpyrrolidone and react with silk fibroin macromolecules. The entangled molecular chains form an interpenetrating network hydrogel, with random coil structures being the dominant ones. The silk fibroin hydrogel is strong, elastic and transparent, which makes it widely applicable as a polymer material for corneal repair.

The preparation method of the injectable silk fibrin porous hydrogel of patent CN109851819B simulates the molding process of natural silk without adding any crosslinker [39]. It consists of preparing an aqueous silk fibroin solution, stirring and shearing to concentrate the silk fibroin. The silk fibroin fluid foam, which is obtained via pre-crosslinking, is injected into a mold and obtained after standing still. Before injection, it can be mixed with specific drugs and biologically active substances to form a hydrogel drug sustained release system. Thus prepared, the hydrogel can be used to fill defects, meet the complex shapes of different wounds and reduce the negative impact of implants on body tissues.

Patent CN110064077B provides a silk fibroin hydrogel which include silk fibroin, poloxamer, cyclic dipeptide, liquid fluorocarbon and indocyanine green [40]. Indocyanine green has a photothermal effect, and liquid fluorocarbons can be quickly converted into gases under the action of 785 nm near-infrared light for intrauterine irradiation and form an elastic three-dimensional porous-structured hydrogel.

Granted patent CN110305339B protects a silk fibroin conductive hydrogel designed to overcome the defects of weak conductive effect and low tensile strength and a preparation method thereof [41]. By changing the secondary structure, the degradation and mechanical properties of silk fibroin materials could be regulated to meet the requirements of various tissues and organs’ needs. A preparation method comprises eight steps which include the preparation of carboxylate silk fibroin powder, polyphenol-modified graphene nanosheets, graphene/silk fibroin polymerization solution, silk fibroin-host solution and silk fibroin-guest solution, adding horseradish peroxidase.

Patent CN112316219B discloses an anti-adhesion hydrogel–silk scaffold composite film that is prepared using the following method [42]: the silk fibrous web was immersed in the hydrogel precursor (acrylamide, *N,N′*-methylenebisacrylamide, CaCl_2_, sodium alginate, ammonium persulfate and tetramethylethylenediamine in deionized water), then sealed and finally thermally polymerized at room temperature. The anti-adhesion hydrogel–silk scaffold composite film is beneficial to mass production since it has no biological toxicity and possesses anti-cell adhesion and excellent mechanical properties; it has good biocompatibility and great application prospects.

In summary, silk fibroin, as a high-quality natural fiber, has been successfully designed from simple crosslinked structures to functionalized crosslinked hydrogel forms (from traditional to smart hydrogels and composite materials). Based on its advantages and particular biological features (biodegradability, biocompatibility, non-immunogenicity, stability, insolubility), it has many biomedical applications (e.g., as a scaffold for growing tissue grafts, bone, corneal and skin regeneration, in controlled drug release and wound healing). Silk fibroin’s main disadvantages are its fragile texture, low tensile strength and mechanical features. Many inventors found that the functionalization of silk-based hydrogels provides high-quality performance and features and compensates for their lack of mechanical characteristics. Inventors found methods for the optimization of its molecular structure by integrating the features of various materials. Additionally, conductive hydrogel was invented in order to overcome the defects of weak conductive effect and low tensile strength. By changing the secondary structure, the degradation of silk fibroin materials may be regulated to improve features for the needs of different tissues and organs. Some different, inventive approaches to overcoming disadvantages are presented in the analyzed patents. One of many advantages of silk fibroin is its functionalization, which suggests a possible solutions for its development and improvement.

#### 2.1.3. Collagen

Collagen is a biopolymer based on a trimeric molecule, which is composed of three intertwined alpha-helices [43]. Its structure was first discovered in 1940 and since 1955, after several years of research, the advanced and refined triple-helical structure has been accepted [44]. Collagen has immense tensile strength due to hydrogen bonds inside its triple-helix structure. It has a cationic flexible polymer structure, which contains primarily hydrophobic peptide motifs. It is regarded as the main structural protein in vertebrates. As the main structural protein in the extracellular matrix, it was found in different connective bodies’ tissues. Its basic function in the extracellular matrix is to supply constructional support. This function, as well as its comprehensible organization with other biological categories, low antigenicity and immunoreactions, excellent biocompatibility, biodegradability and polyelectrolyte behavior, makes it a useful material for scaffolding [5,45]. Collagen is one of the most frequently used biopolymers for biomedical research and cell cultures. Main collagen´s advantages are: good cell adhesion substrate, weak immune response [46], and chemostatic [47]. It can be effortlessly transformed and degraded by cells. Its chemical crosslinking reduces degradation and enhances extended mechanical features. Collagen has application for corneal substitutes, wound healing [48], bone tissue engineering [49,50] and also in the food industry [51].

A total of 6650 patent applications were found in the Espacenet database, filtered according to the keyword “collagen” in the title, abstract and claims during period from 1953 to 27 May 2023 [13]. In order to narrow this search, documents which only had the keyword in the abstract and claims were excluded. Based on these criteria, 159 patent applications (since 1988) of collagen biopolymer hydrogels were found, out of which 59 patents were granted, and only 3 of them were granted prior to 2013. A summarized selection of some of the relevant patents is presented in Table 3 and additionally described.

Patent rights were granted for a newly obtained method for radiation crosslinking collagen gel by irradiating liquid collagen with a low dose of radiation [52]. The liquid collagen was mixed with Pluronic F-127, PEO and hydroxyapatite, and the mixture was irradiated with γ-rays to crosslinking.

A method for conducting an electrospinning reaction to form collagen fibers were protected by registered patent US10730928B2 [53]. The method includes acidifying a collagen using acidic solvent (pH of about 2 to about 4) to form an acidic collagen solution, its electrospinning within an alkaline atmosphere (e.g., ammonia vapor) to form collagen fibers and collecting the collagen fibers within a salt bath (e.g., including ammonium sulfate).

Composite collagen–hydrogel materials for tissue engineering and implantable ophthalmic devices with incorporated composites were protected by patent EP3393534B1 [54]. This composite collagen–hydrogel material consists of the first collagen network (crosslinked with a first crosslinker), and/or a second collagen network with collagen crosslinked by a second crosslinker and a three-dimensional collagen network with plastically and partially compressed collagen hydrogels with a compression degree of 50–95%. A three-dimensional collagen network is embedded in a first and/or second collagen network and they are physically and chemically linked in the composite collagen–hydrogel material.

Granted patent US11426492B2 presents filler glue based on collagen, corneal implants, and collagen-like peptides (CLP-PEG) [55]. Collagen-like peptides consists of a conjugate of the polypeptide (SEQ ID NO:5 or SEQ ID NO:10) and polyethylene glycol maleimide combined to the peptide motif of SEQ ID NO:14. This patent discloses highly efficacious and robust crosslinked collagen and novel collagen-like peptides, as well as their applications in hydrogel preparation, filler glue and corneal implant.

Patent CN108543115B describes the preparation method of an osteoinductive hydrogel loaded with nano fish bones based on “collagen chemical modification” and “dopamine self-polymerization assembly” biomimetic construction [56]. This method greatly improves the interface compatibility between the particulate bone and organic phase in the prepared hydrogel. Moreover, it makes the prepared hydrogel possess excellent osteoinductive properties and opens up new areas for the high-value transformation of skin collagen (pig skin, cowhide, sheep skin, donkey skin and fish skin type I collagen) and fish bone.

The goal of patent CN110124113B is to provide an oriented conductive collagen hydrogel as well as its preparation method [57]. It has an oriented microstructure, possessing stable physical, chemical and conductive properties. Additionally, it has good biocompatibility, which can carry out in situ simple and efficient three-dimensional cells packaging, which can simulate the bionic construction of nerve tissue scaffold.

Patent KR102119693B1 protects a method of obtaining a succinate composite hydrogel based on fibrinogen and collagen [58]. The novelty of this process is the prevention of collagen flocculation in a simple process, improvement of hydrophilicity, a cell affinity, cell proliferation rate and cell diffusion performance. Produced collagen-based biomaterial has excellent biocompatibility.

A method for obtaining a temperature-sensitive collagen-based hydrogel loaded with biologically active polypeptides for repairing bone and articular cartilage defects is protected with patent CN111184917B [59]. This method is characterized by several steps including dissolving N-vinylcaprolactam, ammonium persulfate and recombinant human type III collagen in deionized water, the mixing of this aqueous solution with methacrylic acid, dialyzing and lyophilizing steps and the preparation of poly(lactide-co-glycolide) microspheres containing biologically active peptides. The recombinant human type III collagen applied was obtained via the genetic engineering of yeast fermentation. The obtained hydrogel has non-immunogenicity, injectability, good biocompatibility and degradability.

A method for preparing hydrogel by using collagen S-VCL-S and hydrogen peroxide crosslinked over disulfide bonds was the subject of patent CN112521491B [60]. Collagen sequence design connected the gene fragment to the pET-28a plasmid. It is applied as the carrier for hydrophilic drugs slow-release, has redox responsiveness and can undergo molecular phase transition in response to H_2_O_2_ oxidation. Additionally, it is used to prepare cell scaffold material, which can support cells’ adhesion and proliferation.

Patent CN112717200B protects a method of preparation of an absorbable hydrogel skin repair scaffold based on the recombinant human type III collagen, crosslinked by 1-(3)-dimethylaminopropyl)-3-ethylcarbodiimide hydrochloride and N-hydroxysuccinimide, with the antibacterial component (sodium benzoate, benzalkonium bromide, methylparaben or paraben and at least one of methyl ester, polyethylene glycol and polyhexamethylene biguanide hydrochloride) [61]. This recombinant human collagen absorbable hydrogel skin scaffold has excellent water absorption, water retention and air permeability and can ensure moist restoration conditions for the wound. Furthermore, it directly supplies the skin with the same material as the human collagen that is needed for cell repair, directly participates in the remodeling process of the extracellular matrix of the skin dermis and causes scars reduction.

Collagen is one of the first biobased materials which was used in the field of bioengineering because of its numerous advantages (immense tensile strength, low antigenicity and immunoreactions, excellent biocompatibility, biodegradability and polyelectrolyte behavior, weak immune response). Collagen’s disadvantages, such as its fast degradation rate, high shrinkage, weak mechanical strength and opacity represent the limitations of its usage. Modification of collagen to improve its mechanical features (compliance, elasticity, strength) can further expand its usage. Collagen-based hydrogels have been designed in recent years by using inventive strategies for their functionalization, and some approaches in the granted patents are analyzed in this review (e.g., radiation crosslinking collagen gel, electrospinning to form collagen fibers, osteoinductive hydrogel loaded with nano fish bones, temperature-sensitive collagen-based hydrogel loaded with polypeptides, carrier for hydrophilic drugs slow-release, cell scaffold material for cells adhesion and proliferation support). Finally, the handling of collagen hydrogels, the scalability, full control over the drug release kinetics, shelf life and related analyses need to be upgraded to obtain the safe biomaterial of choice.

#### 2.1.4. Keratin

**Figure 2:**
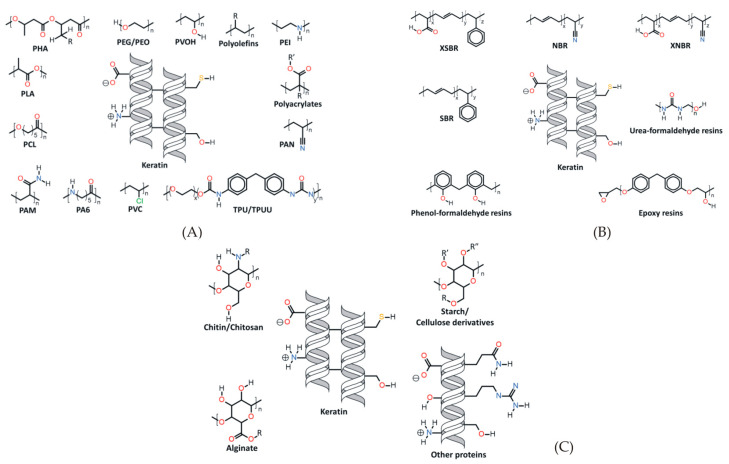
Schematic representation of (**A**) the synthetic and biosynthetic polymers discussed, evidencing their available functionalities for interacting with keratin; (**B**) the elastomers and thermoset polymers discussed, evidencing their generalized structures and available functionalities for interacting with keratin; (**C**) the natural polymers discussed (carbohydrates and proteins), evidencing their generalized structures and available functionalities for interacting with keratin. Reprinted from ref. [64] under open access creative common CC-BY license.

Keratin presents one of the most abundant animal proteins in nature. It can be extracted from the epidermal structure of animal hair, hair, nails or feathers through oxidation and reduction methods. Animal hair contains a large amount of keratin, and its content can reach more than 95%. Keratin has been applied both as a structural and a biomedical substance for centuries, but it was rarely studied because of its difficult extraction process. The investigation of keratins structures began approximately eight decades ago [2]. The progress of extraction processes largely contributed to the advancement in keratin applications in a renewable manner, particularly regarding contemporary methods without harmful solvents (e.g., steam explosion or ionic liquid-based extractions) [62]. There are many studies on the cross-modification of keratin and synthetic polymers to prepare biomaterials [63]. It has excellent biocompatibility, biodegradability and low cytotoxicity, and it has the potential to mold a definite three-dimensional microstructure. It upholds the proliferation and infiltration of cells and tissue formation guided by cells, making keratin suitable for use in the biomedical field. Keratin proteins are particularly explored for use in the preparation of useful materials for biomedical usage. Representation of keratin’s generalized structures and accessible functional groups for interaction with synthetic and biosynthetic polymers, elastomers and thermoset polymers and natural polymers (carbohydrates and protein) were discussed in a review by Donato et al. (Figure 2) [64].

Keratin solutions can be converted into fibrous three-dimensional scaffolds using the electrospinning method [65]. Because of their distinctive capability of self-assembly and polymerization, they were used as porous reproducible ultrafine keratin fibers built for controlled cell growth [66]. Successful keratin applications in functional biomaterials production are extensive and diverse, ranging from usage in pharmaceuticals as drug carriers [67,68], for nerve regeneration [69], as biopolymer absorbents [70] and in agriculture [71].

In the Espacenet database, 1221 patent documents were found, which were filtered according to title, abstract and claims for biopolymer hydrogels based on keratin in the period from 1915 to May 2023 [13]. In order to narrow this search, instances of the keyword in the abstract and claims only were excluded. Keratin as a biopolymer hydrogel was the subject of 29 patent applications (since 1996), 11 of which have been registered. For the last ten years (from 2013 to 2023), 11 patents were granted. Selected documents are summarized in Table 4 and described below.

Keratin-based hydrogels and aqueous sterile, injectable compositions (comprising living cells and the second bio polymer, e.g., alginate, chitosan or gelatin) for application in tissue regeneration, with the relevant obtaining methods, were protected by patent US10723774B2 [72]. The crosslinking functionality is bonded to the keratin over cysteines after the disulfide bonds reduction of the native keratin by a photopolymerizable crosslinking moiety using ultraviolet radiation, visible light or infrared radiation.

The object of patent CN107828031B is to provide an aqueous urethane acrylate grafted keratin hydrogel, which is good at absorbing heavy metal ions [73]. A method for obtaining aqueous polyurethane acrylate grafted keratin hydrogel sourced from degreased pig hair via the use of petroleum ether as the solvent and azobisisobutimidazoline hydrochloride as a photoinitiator was described.

Patent CN110511405B belongs to the field of biomass materials [74]. The preparation method includes the simple reaction of grafting alkenyl quaternary ammonium salt, a fast synthesis rate, mild conditions, high yield, simple separation and purification. Oligomers generated in the free radical-mediated reaction process were avoided. The obtained grafted keratin still preserves its gel-forming properties, and the prepared hydrogel has good antibacterial properties against both Gram-positive and Gram-negative bacteria.

Patent CN111825858B discloses a composite hydrogel based on zwitterions and keratin with excellent degradability and excellent biocompatibility [75]. The synthesis method is simple. The zwitterionic degradable hydrogel is prepared by free radical polymerization and it could be applied in the biomedical field as an anti-adhesion wound dressing.

The preparation method of patent CN113354840B is quite simple; it adopts the freeze–thaw cycle method without other chemical crosslinkers [76]. Low-temperature treatment prevents denaturation of keratin. At the same time, the binding forces between keratin and between keratin and water molecules is balanced. Obtained keratin hydrogel is cheap, non-toxic, biodegradable and environmentally friendly, with high transparency. The application of external coating materials rabbit hair keratin in the biomedical field not only broadens the application but also improves the reuse value of waste natural polymer materials, thus reducing biomass waste.

Keratin is one of the toughest natural materials, regardless of the fact that it is protein; it shows great potential for application in innovative, bioinspired strategies and biopolymers because of its excellent biocompatibility, biodegradability, low cytotoxicity, high mechanical strength and compact biological features. Keratins have assorted hierarchal structures, a porous network and high chemical reactivity after alteration and have the potential to mold a definite three-dimensional microstructure. The presented innovative methods and applications in this review include conversion into fibrous three-dimensional scaffolds via the electrospinning method for usage in controlled cell growth, as drug carriers, for nerve regeneration, as biopolymer absorbents, in tissue regeneration, or as heavy metal ions absorbents. Keratin is modified by the second biopolymers (e.g., alginate, chitosan, or gelatin) or synthetic polymers (e.g., polyurethane, acrylate). Many additional research investigations are needed in order to understand the importance of keratin-based biomaterials, which could be useful in new biomedical applications.

#### 2.1.5. Gelatin

Gelatin is a type of protein obtained using the controlled, partial, irreversible hydrolysis of collagen, which is extracted from boiled animal tissues (e.g., bone, skin and cartilage)—usually from fish, bovine or porcine [77]. Depending on the process used and the types and ages of the animals, generally, two gelatin types are derived, namely, type A (by acid hydrolysis) and type B (by alkaline hydrolysis). Triple-helical conformation, which is inherent of collagen, is partially denatured, and the obtained gelatin is mainly amorphous. Because single chains have reduced molecular weight, the resulting gelatin has a high polydispersity. Numerous gelatin lateral functional groups provide suitable mechanical characteristics by additional chemical crosslinking [78].

**Figure 3:**
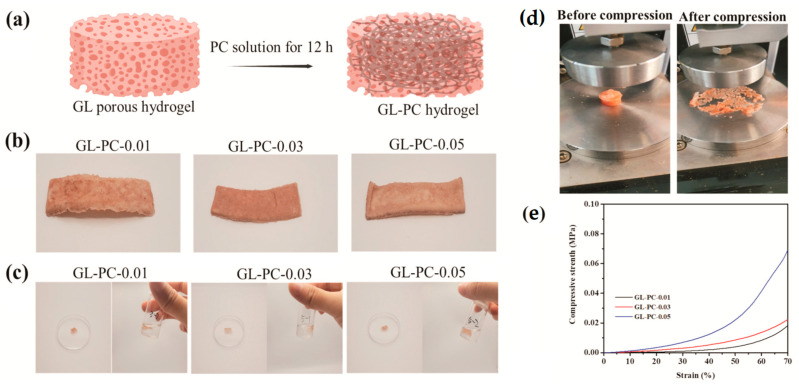
(**a**) Schematic representation of preparation of GL-PC porous hydrogels for cultivated meat production. (**b**) Digital photograph images of GL-PC hydrogels after freeze-drying. (**c**) Digital photograph images of the stability of GL-PC hydrogels in PBS (pH 7.4) at 37 °C. (**d**) Digital photographs of GL-PC hydrogels before and after compression. (**e**) Compressive analysis of GL-PC hydrogels. Reprinted from ref. [88] under open access creative common CC-BY license.

Gelatin is a sequence mixture of peptides. It is soluble in warm aqueous solutions but maintains, at low temperatures, the capability to build simple gels structure by hydrophobic crosslinking. Gelatin, as a biocompatible and non-immunogenic protein due to its unique (physical and chemical) nature, has been applied as a drug and cell carrier. The gelatin melting temperature (from 30 to 35 °C) is a limiting factor for its application at physiological body temperatures or higher. Due to this limitation, it is usually chemically modified in many inventive ways, e.g., by additional crosslinking processes. It is famous for its applications in the food industry [79,80], and it is also extensively applied in the textile and pharmaceutical industries because of its capability to build flexible, inexpensive and thermoreversible gels. Gelatin-based hydrogels are non-immunogenic, non-toxic and water-soluble materials. Because of their exceptional biocompatibility and biodegradability in physiological conditions, they were employed for various biomedical uses, such as for cell encapsulation, wound healing [81,82], skin substitute [83], regeneration of nerve [84], reconstruction of soft tissue [85], bone repair [86] and 3D bioprinting [87]. An interesting study was presented wherein cell-cultured artificial meat was obtained using bovine satellite muscle-derived cells cultivated in vitro, which were capable of growing and connecting to a porous naturally occurring gelatin (GL)-based hydrogel enriched by proanthocyanidins (PC) from grape seed extract (GL-PC) (Figure 3a) [88]. The compressive strength values for GL-PC samples (1.12, 1.36 and 1.41 kPa) notably enhanced the enlarging content of proanthocyanidins in hydrogels because of the enhanced complexation among GL and proanthocyanidins, and they were similar to those of bovine muscle (1.2–1.8 kPa) under the same strain (Figure 3b).

In the Espacenet database, 6610 patent documents were found, filtered according to title, abstract and claims for collagen biopolymer hydrogels in the period from 1953 to May 2023 [13]. Obtained data were additionally filtered in order to include only documents with the keyword “gelatin” in the title and an earliest priority date after 2013. Based on collagen biopolymer hydrogels, there are 140 patent applications found (since 1988), 58 of which were granted patents and only 4 prior to 2013. A summarized selection of some of the newest relevant granted patents is presented in Table 5 and additionally described below.

A crosslinked gelatin hydrogel with a high crosslinking density and a high mechanical strength or a gelatin derivative for producing the porous body was described in granted patent JP7050296B2 [89]. The amino group possessed by gelatin is bound to a methacryloyl group, and the hydroxy group and the carboxyl group possessed by gelatin are bound to a methacryloyl glyceryl ester group. The gelatin was extracted from the group of animal bone, animal skin, fish bone, fish skin and fish scale.

The essence of patent PL237373B1 is the application of L-3,4-dihydroxyphenylalanine immobilized in gelatin hydrogel matrices for the production of a colored test for the detection of polyphenol oxidase (tyrosinase), particularly in aqueous solutions [90]. L-3,4-dihydroxyphenylalanine (L-DOPA) immobilized in hydrogel matrices exhibits the capability to transform to a colored product in the presence of polyphenol oxidase (tyrosinase). Therefore, it is possible to selectively detect tyrosinase in, e.g., liquid microbiological cultures or plant extracts, which generally contain a complex mixture of proteins with different catalytic properties.

Patent CN111632189B provides an injectable hydrogel hemostatic agent based on marine source gelatin and the appropriate method of its application. Injection curing using ultraviolet radiation can achieve the effect within 20 s and manifests strong mechanical properties and tissue adhesion [91]. It solves the problem of turn down of mechanical characteristics and sealing breakdown, as well as further harm to the surrounding tissue (i.e., of human or animal body tissue or organ accidental trauma or surgical wounds). Because of the degradable properties of obtained hydrogel, it will gradually degrade as the wound heals. It also has a good biosafety record.

The subject of granted patent CN113230448B is a gelatin-based low-temperature injection anti-inflammatory and antibacterial viscous hydrogel based on gelatin, hydroxyethyl urea and punicalagin, as well as its preparation method [92]. The hydrogen bond interaction between gelatin and hydroxyethyl urea is used to obtain low temperature injection; later, punicalagin is added, and the strong hydrogen bond interactions between punicalagin and gelatin are built to form a main network, which is stimulated by medical alcohol.

The aim of patent CN113637187B is a new method for the preparation and application of a methacrylic gelatin hydrogel with grafted photoinitiator molecules [93]. The first step is preparation of 2-hydroxy-4′-(2-hydroxyethoxy)-2-methylpropiophenone-acrylated photoinitiators in a solvent. They are formed by dichloromethane and triethylamine, and then acryloyl chloride is added to the mixed solution without light to obtain the acrylated photoinitiator. The second step is the preparation of methacryl-based gelatin by grafting photoinitiator molecules. The final step is the preparation of methacryl-based gelatin hydrogels grafted with photoinitiator, which were dissolved in ultrapure water and irradiated with ultraviolet light.

The goal of patent CN114213682B is the preparation method and application of a gelatin-based glycolipid hydrogel with double dynamic crosslinking [94]. The present invention utilizes the excellent biocompatibility of gelatin and the relatively low crosslinking cost to prepare a dynamically crosslinked gelatin-based glycolipid hydrogel through Schiff base crosslinking and coordination crosslinking. The obtained gelatin-based hydrogel has excellent adhesive, mechanical, self-healing and injectable properties, with a potential application in wound repair.

Based on various advantages (biocompatibility, biodegradability, swelling capacity, non-immunogenicity, self-healing, commercial availability) gelatin is used in the medicine, food and pharmaceutical industries. Due to its many advantages, it became an attractive biomaterial in many fields of usage (like cell encapsulation; wound healing, skin substitute, regeneration of nerves, reconstruction of soft tissue and bone repair, as well as 3D bioprinting, etc. Principal weaknesses of gelatin-based hydrogels are their poor mechanical strength and low melting temperature. Over the years, scientists and inventors have developed different variations of gelatin and its modified hydrogels; some of the possibilities include combining gelatin with other natural and/or synthetic polymers (with different mechanical features) as well as various obtaining methods (e.g., copolymerization, grafting) in order to overcome its weaknesses for potential applications. Contemporary advancements in this field will include the influx of new generation of effective biomaterials available for biomedical usages.

### 2.2. Polysaccharides

Polysaccharides are large molecules consisting of many simple sugars as monosaccharide units which are covalently bonded via glycosidic linkages by special enzymes creating large sugar polymers. Polysaccharides could be separated into homopolysaccharides (the monosaccharide units are the same) and heteropolysaccharides (the monosaccharides units are different). Polysaccharides could have a linear (usually packed in a rigid structure) or branched (mainly soluble in water) molecular structure. They are important materials of living things as they are crucial to the structural support of cells and organs, as well as to energy storage within the organism [95]. Polysaccharides have the potential to be prosperous biopolymers because of their high stability and low cytotoxicity [96], and for this review, chitosan, hyaluronic acid, alginate, carrageenan and cellulose were analyzed.

#### 2.2.1. Chitosan

Chitosan is a copolymer composed of a random distribution of units of N-acetyl-D-glucosamine and linear β-1,4-D-glucosamine jointly via β-(1→4) glycosidic linkages [97]. It belongs to the family of naturally available polysaccharides, as the only known cationic polymer of natural origin (positively charged polyelectrolyte). It is characterized by the deacetylation degree that presents the percentage of repeating units of glucosamine in its macromolecular chain. Chitosan is obtained by fully or partial deacetylation of chitin or extracted directly from fungi. Chitin is a constituent of arthropods’ (crustaceans) exoskeletons or the invertebrate endoskeletons of cephalopods. It has molecular weights from 50 kDa to 2000 kDa. Chitosan has a high content of amine functions (of the order of 5 mmol per gram), depending on the degree of deacetylation (which can be in the range of 40–98%, usually between 60% and 90%). Chitosan is a polymer available in the form of powder or flakes with larger or smaller particle sizes. It is the only natural amino polymer in powder form, insoluble in pure water but soluble in acidified water with at least 1% v/v concentrated acetic acid. Because of the presence of ionic forces, an aqueous chitosan solution alkalized to a pH value over 6.2 causes hydrogel precipitation. As a promising biomaterial candidate, it is broadly applied in biomedicine because of the biocompatibility, bioresorbability, hemocompatibility, healing and anti-growth properties of bacterial strains. However, it possess weak mechanical performance and is unsuitable for application in hard tissue engineering. Scientists have investigated some techniques for chitosan modification, e.g., to strengthen the properties of chitosan-based bio-inks [98]. Chitosan has very good antimicrobial and antioxidant characteristics.

Hydrogels based on chitosan have been broadly investigated with respect to many biomedical application, mostly as drug delivery systems [99] and for wound dressings [100], cartilage tissue engineering [101], skin regeneration [102] and bone regeneration [103]. Chitosan-based hydrogels films have been applied to preserve vegetables, fruits and meat in food packaging industries [104]. Additionally, encapsulation of chitosan-based enzymes was studied for usage in the food industry [105].

Patent documents were researched using the advanced search option by title, abstract and claims in the worldwide Espacenet database. Based on the defined criteria, 7088 documents (granted patents and patent applications) were found from 1975 to May 2023, focussing on chitosan-based biopolymer hydrogels, and then narrowed down to involve only patents with the keyword “chitosan” in the title and a priority date after 2010 [13]. Chitosan as a biopolymer hydrogel was the subject of 484 patent applications (since 1995), of which 209 have been registered patents. In the last ten years (from 2013 to 2023), 182 patents have been granted, and some of the selected documents are summarized in Table 6 and described below.

The purpose of patent CN112940287B is to optimize the preparation method of conventional chitosan cryogel with improved mechanical properties and excellent water absorption and to provide a chitosan hydrogel with a shape memory function [106]. The deprotonation of glycans was changed from a solution to a gel under the action of a strong shearing force. Molecular chains of chitosan were assembled to obtain chitosan fiber bundles (both diameter and length about 50 nm) with good stability within a few μm. Chemical crosslinking improved chitosan’s properties, e.g., acid-resistance and mechanical properties (super-elastic feature, cyclic compression fatigue resistance, high plasticity). Dehydrated chitosan cryogel can basically recover its shape after 3 s rehydration.

Granted patent EP2538987B1 protects the method of production of a hydrogel matrix based on cartilage-forming cells. Cartilage-forming cells, alginate (1–1.4%) and chitosan (0.5–0.7%) (Mw lower than 60 kDa) are mixed and then polymerized into spherical hydrogel beads with a diameter between 0.01–5 mm for use in the repair of cartilage defects [107].

Patent EP2874672B1 relates to the usage of chitosan in nerve cell regeneration, in the repairing of the nervous system (preferably the central nerve system), in nerve cell grafting, in the treatment of paralysis and/or in the treatment of neurodegenerative diseases [108]. The applied chitosan has an acetylation degree no higher than 20%, and the concentration thereof in the hydrogel is 0.25–5% with respect to the whole hydrogel weight. Additionally, this patent disclosed an implant including a suspension of microparticles mixed with water, stem cells and/or trophic factors and/or Schwann cells.

The subject of patent US9814779B2 is a copolymer hydrogel which comprises a copolymer of crosslinked chitosan, polylactide, fibrinogen, hydrolysable methacrylate crosslinker and one or more absorbed bioactive agents [109]. This crosslinked copolymer hydrogel is composed for the sustained release of bioactive agents (e.g., bone morphogenetic protein-2, bovine serum albumin, amniotic human mesenchymal stem cells cardiac repair and regeneration) at a first-order release rate.

The subject of the protected patent EP2920240B1 is the aqueous solution of chitosan, the method of its acquisition and its usage in the form of chitosan aerosol and chitosan composition [110]. A chitosan hydrogel membrane obtaining method was also disclosed, as well as a chitosan-protein obtaining method. The obtained hydrogel membrane showed effective antibacterial activity against *Staphylococcus aureus*.

Chitosan in the hydrogel, according to patent EP3317326B1, has a role in providing the architecture of the gel with the required rheological properties [111]. These hydrogels are biocompatible and advantageously thixotropic, which allows for their injection with a syringe. Once the hydrogel is placed in the body of the syringe, it liquefies under shear stress applied across the plunger to flow through the exit port of the syringe into the needle, and possibly into the catheter. Once released at the site of application, it recovers to form a solid hydrogel for at least several hours and/or does not flow and remains at the site of application to release one or more active substances, i.e., ciprofloxacin.

Patent EP3412313B1 provides a temperature-sensitive hydrogel composition including chitosan and nucleic acid. The obtained hydrogel has an exceptional biocompatibility, biostability and, simultaneously, sol–gel phase transition depending on the temperature [112]. At room temperature, this hydrogel is in the sol state; when the temperature increases, it becomes a gel, e.g., when it is applied on an epithelial skin surface or injected into the body at specified area in need of healing. Drug attaching and retention time is enhanced during gelation since these processes depend on the temperature. Drug efficacy is satisfactorily manifested, and it could be applied for various treatments.

The subjects of granted patent US11161958B2 are macroporous polymeric hydrogel microspheres based on chitosan and polyacrylamide. The average pore size ranges from 1 to 60 nm, with a diameter of 50–250 μm [113]. The hydrogel microspheres are able to carry conjugated biomolecules. Its production method comprised photo-induced polymerization and the micromolding technique via surface tension-induced droplet formation.

Granted patent US10828319B2 describes a method of preventing intramammary infection and the acceleration of regressive changes via application of a chitosan solution as a biological response modifier to the teat of a lactating mammal during drying-off [114]. A neutralized chitosan solution using β-glycerophosphate for injection is in a liquid state at room temperature. At body temperature after injection, it forms a hydrogel inside the teat.

Patent EP3765104B1 relates to a hydrogel composite obtainable via the gelation of a suspension based on chitosan (hydroalcoholic or aqueous), dispersed TEMPO-oxidized cellulose nanofibers and an acid [115]. The suspension is convenient for regeneration of tissue and as biocompatible and bioresorbable knitted textile implant.

Patent CN110237782B discloses a method for obtaining an anti-oxidation composite hydrogel based on chitosan/polydopamine with good mechanical properties and antibacterial activity [116]. It is biodegradable, non-toxic, safe and harmless. The production process is simple; it consists of a chitosan added to the solvent (mixed aqueous solution of LiOH, KOH and urea) to obtain a chitosan alkaline solution via freezing and thawing. The composite hydrogel could be applicable in the fields of cosmetics, medicine and environmental protection.

Patent KR102372964B1 relates to a chitosan-derived hydrogel sensitive to near-infrared ray, a method of its acquisition via a click reaction and its application as a drug delivery system [117]. After body administration, the drug release can be actively controlled by controlling the amount of near-infrared radiation from outside and can be usefully applied in the pharmaceutical and cosmetic industries.

Chitosan is a promising biomaterial with extensive use possibilities (e.g., in drug delivery systems, for skin, nerve cell or bone regenerations, tissue engineering, films for food packaging, cosmetics). The weak mechanical features of chitosan hydrogels comprise the principal disadvantages for its application, especially in tissue engineering. Additionally, they showed a mismatch in the degradation rate and low cell adhesion. Many researchers and inventors have applied the available methods and inventive strategies for molecular structure modification in order to compensate for these weaknesses and enhance the quality and features of chitosan-based hydrogels. Some approaches in the granted patents analyzed in this review include copolymerization with polylactide or fibrinogen, using hydrolysable methacrylate as a crosslinker, copolymerization with monomers sensitive to temperature or to infrared rays, hydrogel composition in microsphere form, designing the required rheological features and employing shape memory function, click reaction method or similar. Additional attentive evaluations of the relevant properties of chitosan-based hydrogels and the in vivo safety of formulations are important for future usage and final product registration.

#### 2.2.2. Hyaluronic Acid

Hyaluronic acid is a polysaccharide consisting of a non-branched, linear structure. It is an anionic copolymer composed of *N*-acetyl-β-D-glucosamine and β-D-glucuronic acid, interconnected by alternating glycosidic bonds (1→3) and (1→4) with molecular weights in the range of 103 kDa–104 kDa [118]. Hyaluronic acid naturally originates from the extracellular matrix of cartilage and synovia. It provides joint protection by supporting the viscosity of the synovia and forms more elastic joint cartilage. Hyaluronic acid is found naturally in different parts of the body (e.g., connective tissue, cartilage extracellular matrix, umbilical cord, vitreous humor, skin, joints and synovia) and is omnipresent in vertebrates [119]. Concerning its mechanical features, a single molecule of hyaluronic acid exhibits viscoelasticity depending on the ionic strength and pH value of its environmental [120]. Hyaluronic acid exhibits low immunogenicity and excellent biocompatibility. In order to enhance its mechanical features and build a hardy biomaterial, some approaches consisted of chemical modification, e.g., by numerous functional groups, or by crosslinking.

The modified hyaluronic acid hydrogels showed considerable potential in many applications in regenerative medicine [121,122], cosmetology [123] and wound healing [124], as well as in bone [125] and cartilage repair [126] and 3D bioprinting [127].

In the worldwide Espacenet database, based on the defined criteria, 5048 patent documents were found (patent applications and granted patents), filtered using advanced search option by title, abstract and claims from 1986 to 27 May 2023 [13] for biopolymer hydrogels based on hyaluronic acid. Then, the search was narrowed to involve only patents with the keyword “hyaluronic acid” in title, and earliest priority date was set to 2012. Hyaluronic acid as a biopolymer hydrogel was the subject of 236 patent applications (since 1993), of which 107 are registered patents. In the past ten years (from 2013 to 2023), 96 patents were granted according to the Espacenet database, and some of the selected documents were summarized and described in Table 7.

Patent KR101953709B1 relates to a new hyaluronan derivative, along with the method of its obtaining. This patent also covers a new hydrogel derivative, the method for obtaining method this hydrogel and the uses of thus hydrogel in cosmetics, medicine, tissue engineering, regenerative medicine and, in particular, in the form of scaffolds for articular cartilage or bone tissue defect treatments [128].

Granted patent EP3007737B1 describes a procedure for hyaluronic acid crosslinking and/or its sodium, calcium, zinc or potassium salts, as well as procedure for the preparation of sterile, injectable hydrogel [129]. Obtained hydrogels comprising active substances with or without pharmaceutical activity (e.g., anticancer agents, antioxidants, antifungal agents, antibacterial agents, anti-inflammatories, antiseptics, anesthetics-lidocaine, proteins, biological entities, hormones, single or in a composition) are disclosed, as are their uses in therapy and in non-therapeutic and non-surgical esthetic applications.

The object of patent EP3049055B1 is the preparation method for the sterile, injectable hydrogel, consisting of hyaluronic acid or a salt thereof and lidocaine (combined with alkali), without notably changing the required characteristics [130]. This method includes several consecutive steps. The pH value is between 6.5 and 7.6. Bifunctional or multifunctional crosslinker molecules were selected from epoxides, epihalohydrin and divinyl sulfone.

Patent EP3139961B1 protects a pharmaceutical composition which includes a hyaluronic acid derivative hydrogel loaded with at least one exogenous enzyme (prolyl endopeptidase, endoprotease and combinations thereof). The composition is proposed for oral treatment of celiac disease [131].

Patent KR101838715B1 discloses microstructures of crosslinked hyaluronic acid hydrogels with a uniform shape and minimum deformation, as well as the method by which it is obtained [132]. They could be used to slow down skin aging, e.g., to repair wrinkles, due to the fact that they easily absorb body fluids and replenish moisture because of their remarkable swelling capacity. Additionally, the obtained microstructures ensure long-lasting features in the inner body based on their resistance to a hydrolyzing enzyme, and they enable safe delivery of beneficial constituents in the human tissue.

The subjects of patent US10300169B2 are hydrogels comprising a macromolecular network based on a crosslinked hyaluronic acid and silk fibroin via a multiamine crosslinker (lysine methyl ester) and water. The obtained macromolecular matrix could be applied to enhance human soft tissue, create space in tissue and support cell or tissue viability or proliferation [133]. The mentioned patent also protects a method of grafting fat (comprising a lipoaspirate) onto the composition, based on a crosslinked macromolecular matrix and water in a human body. The novelty of this invention is the possibility to produce a uniform microstructure of crosslinked hyaluronic acid hydrogel with minimized distortion. The obtained microstructure hydrogel showed improvements in wrinkles correction and ensures a moisturizing effect. The hydrogel effortlessly absorbs body liquid because of its excellent swelling capacity, and it is capable of delivering loaded components due to hyaluronidase stability and its long duration within the living body.

The subjects of patent KR102099981B1 [134] hydrogels that could be applied to enlarge soft human tissue, support or promote the proliferation or viability of tissues or cells and form space in the soft tissue. The obtained matrix platform used hyaluronic acid conjugated to a pyrogallol functional group. This conjugate can be promptly crosslinked by two different methods, i.e., by adjusting pH or an oxidant. It can efficiently control physical features, e.g., adhesive power, crosslinking speed and elasticity, and possesses excellent biocompatibility. The hydrogel is applicable in drug delivery, as either an anti-adhesive or a wound-healing agent, and also in cosmetic products.

The subject of patent KR102334794B1 is a filler based on a hyaluronic acid hydrogel, characterized by elasticity and cohesion as filler performance indicators and with excellent features such as tissue repair ability and wrinkle improvement [135]. It is able to maintain shape for a long time and it is less likely to detach from the injection site, having low crosslinking agent content.

Patent KR102398680B1 protects a specific biomaterial via its filling property, innovated using the microrheology method, and shows enhanced characteristics (e.g., high viscoelastic flow). Additionally, it shows low movability after percutaneous administration while still maintaining its shape and manifests excellent soft tissue repairing features, excellent volume expansion (for the lips, breasts, cheeks, nose or bottom) and wrinkle alleviation [136].

The hydrogel containing serotonin-modified hyaluronic acid, disclosed in patent CN113574109B, can be obtained via adjustment to the crosslinking ratio, and its physical properties are obtained by adjusting the oxidation conditions [137]. It is used as a hemostatic composition for stopping bleeding by promoting blood coagulation. This hydrogel has multiple applications, e.g., as a prevention of tissue adhesion and hemostasis, as a drug delivery system and the promotion of cell differentiation. It has excellent biocompatibility and high availability.

Hyaluronic acid has very interesting features and great potential for biomedical use. Currently, it is commonly applied as an injectable hydrogel in cosmetology and regenerative medicine to enhance human soft tissue due to its elasticity, viscosity and high swelling capacity. Some of its disadvantages include its weak mechanical properties and fast degradation time after injection under living tissue. Known methods and techniques have been applied to enhance its quality, e.g., as mentioned in this review. Well-known methods and techniques and inventive procedures were applied for designing and enhancing its hydrogels quality, e.g., photopolymerization, crosslinking with bifunctional or multifunctional crosslinkers (epoxides, epihalohydrin, divinyl sulfone, multiamine lysine methyl ester, etc.), modulation using the other natural or synthetic monomers/copolymers and the forming of supramolecular structures, as mentioned in this review. Further research is needed with the goal of realizing the maximal potential of hyaluronic acid in its applications as a hydrogel biomaterial in clinics usage.

#### 2.2.3. Alginate

Alginate is a natural polysaccharide originated from alginic acid. It is extracted from cell walls of brown seaweed species (algae class Phaeophyceae). As an anionic copolymer soluble in water, it is composed of α-L-guluronic acid (G) and β-D-mannuronic acid (M) residues, interconnected by α-(1→4) glycosidic bonds. It has convenient biocompatibility, and it is capable of gelation [138]. Alginate has been obtained as a precipitate via treatment of brown seaweed with NaOH and attached to divalent cation (calcium dichloride). Additionally, sodium citrate and ethylenediaminetetraacetic acid as chelators are used for alginate crosslinking. Alginate crosslinking by ionic attractive forces is fully reversible due to the chelation of the used divalent cations. The chemical composition of alginate varies among various algae species and their different parts [139]. More than 200 various alginates forms can be made because of their minute differences in chemical structure, especially their M and G residue ratio in block copolymers [140].

Alginate is applied in the obtaining of biodegradable colloidal structures such as biofilms, nanoparticles, gels, microcapsules and beads, [141], which are convenient for various uses, e.g., in separation technologies, the food industry [142,143], orthodontic use, regenerative medicine [37], tissue engineering [144], drug delivery and wound healing [145].

**Figure 4:**
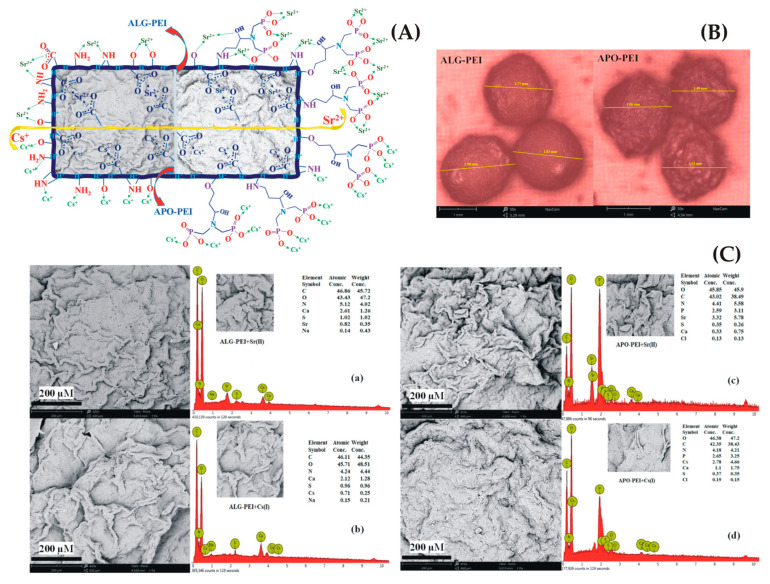
(**A**) Prospective binding mechanisms for Cs(I) and Sr(II) sorption onto ALG-PEI and APO-PEI sorbents. (**B**) SEM photos for shape and size evaluation of sorbent particles. (**C**) SEM observation (left panels) and semi-quantitative EDX analysis (right panels) of ALG-PEI after Sr(II) sorption (**a**) Cs(I) sorption (**b**); APO-PEI after Sr(II) sorption (**c**); and Cs(I) sorption (**d**). Reprinted from ref. [146] under open access creative common CC-BY license.

Alginate hydrogels formation can be in the shape of either beads or fibers. A new process involving pristine branched algal/polyethyleneimine beads (ALG-PEI) was investigated to obtain the APO-PEI beads grafting of phosphonate moieties and was used in the recovery of either cesium, Cs(I) or strontium or Sr(II) with selectivity against other metals (Figure 4) [146]. They were extensively characterized by selectivity with multi-component solutions, uptake kinetics, pH effect, sorption isotherms and desorption of metals (with sorbent recycling).

In the worldwide Espacenet database, 7151 patent applications and granted patents were found, filtered according to title, abstract and claims for alginate biopolymer hydrogels in the period from 1953 to 27 May 2023 [13]. Obtained data were additionally filtered in order to include only documents with the keyword “alginate” in the title and an earliest priority date after 2010. A total of 257 patent application were found (since 1989). During the last ten years, 114 patents were granted, and some of the selected patents are analyzed and summarized in Table 8.

The subjects of patent US9090868B2 are alginate hydrogel fibers and related materials (a kit for implementing biochemical, diagnostic, cellular and non-cellular analysis) with their preparation methods [147]. A three-dimensional cellular order was obtained from an alginate hydrogel paper comprising a majority of alginate hydrogel fibers, which form a non-woven matrix. The alginate hydrogel building up the alginate paper is significantly index-matched with a predesignated culture medium.

Patent EP2688397B1 protects a hydrogel which could be applied to entrap or encapsulate live cells, as well as transporting methods for live cells (entrapped or encapsulated into hydrogels) [148]. The patent discloses enhanced mechanical features associated with strontium alginate hydrogels and reinforced hydrogels (e.g., with nylon meshes). This patent is related to treating methods of wounds, tissue injury or disease (e.g., damaged ocular surface).

Patent EP2916881B1 discloses a method for providing embedded mammalian cells, which includes a phase in which an aqueous solution of sulfated alginate is provided; a gelation phase of its reaction to build a hydrogel; and, in the last phase, the embedding of the precursor cell in the sulfated alginate hydrogel. In such a way, a sulfated alginate hydrogel-embedded cell was yielded [149]. The patent also includes cellular grafts consisting of an embedded mammalian cell in sulfated alginate hydrogel.

Patent EP3086822B1 protects pharmaceutical formulations based on a homogeneous hydrogel consisting of Fibroblast Growth Factor 18, formed in situ [150]. The hydrogels, once formed in situ, could be used for cartilage disorders treatments (e.g., cartilage injury or osteoarthritis). A gelation system was formed from solution 1, comprising alginate, collagen and sugar (as stabilizing agent), while solution 2 comprises a dicationic salt.

The subject of granted patent TWI472536B is the molecular structure modification of the alginate monomer and hydroxyl groups to form carbonyl groups [151]. metal crystallite in a specific size range could be steadily linked into the alginate monomer, alginate salt and alginate hydrogel. The obtained alginate hydrogel with the incorporated metal crystallite is able to deliver metallic atoms and/or ions continuously in a defined pH range, and it could be used in the biomedical, textile and food industries.

Patent EP3433282B1 protects a semi-permeable hydrogel composition based on an alginate matrix covalently crosslinked in the periphery with 3–10 polymer arms [152]. The periphery of the alginate matrix is interlocked with, and covalently crosslinked to, the multi-armed water-soluble polymer. A biocompatible surface layer is covalently bonded to the semi-permeable hydrogel composition. The alginate matrix comprises the pharmacologically active material (living cells, proteins, polynucleotides and small molecules) in a form of a bead, capsule, sheet, membrane, thread, fiber, filament, particle or sponge.

Patent CN110548214B proposed a method for preparing a miniature smart calcium alginate hydrogel, monolayer film terminal and micro-manipulator based on different micro-electrodes to realize different functions [153]. This method includes electrodeposition, treatment, and pick-up.

The object of patent KR102521317B1 is a hydrogel based on an alginate, a methacrylate bonded to a hydroxyl group, an acrylic monomer, and a photoinitiator, characterized by an elastic modulus which increases as the number of uronic acid residues bonded to the methacrylate increases [154]. Producing tetraalkylammonium alginate through cation exchange of sodium alginate includes bonding methacrylate to the alginate hydroxyl group, cation-exchanging the tetraalkylammonium with Na^+^ to obtain meta synthesizing acrylic alginate, mixing methacrylic alginate, an acrylic monomer and a photoinitiator, and photocrosslinking by irradiating UV light.

A hydrogel spraying device and a method for obtaining calcium carbonate solution and alginate solution were disclosed in patent TWI776389B [155]. Hydrogel with antibacterial and moisturizing properties, calcium carbonate solution and alginate solution may be sprayed directly onto the animal’s body surface wound without pain or pressure. This is very suitable as a physiological dressing for animals, especially for pets.

A hydrogel based on a copolymer of oxidized sodium alginate/polyethylene glycol amine was prepared to solve the problem of the poor biocompatibility of the existing hydrogel in 3D cell culture and the lack of corresponding stimulating factors that promote cell proliferation [156]. Patent CN113980294B combines the advantages of sodium alginate as natural active polysaccharide with the biological characteristics of conductive polymers. When combined, they produce a hydrogel with electrical conductivity, self-healing properties and biocompatibility. It conducts cells by simulating a natural extracellular matrix 3D culture. It can realize the important functions of maintaining cell life activities and promoting cell proliferation. The conductive self-healing hydrogel has great potential for the development of new biomedical materials.

Alginate was initially used as an ingredient and additive in the food industry. Due to its properties (biocompatibility, biodegradability, nontoxicity, elasticity, inexpensiveness, etc.), it has been extensively used as a significant biomaterial in many advanced applications in the medical, pharmaceutical and cosmetics industries. Similar to many other natural polymers, alginate has some disadvantages including low mechanical characteristics, poor biocompatibility and dimensional stability. Future investigations into alginate should be directed towards examinations, improvements and the development of new products to overcome its weaknesses. Some of those possibilities have been analyzed in this review. Future development of these biomaterials include technology transfer, innovated production processes, safety and regulatory requirements and users’ acceptability. For the use of alginate in medical or pharmaceutical practices, its consequences for human usage have to be rigorously analyzed in clinical trials.

#### 2.2.4. Carrageenan

Carrageenan is a high-molecular-weight sulfated mucopolysaccharide, originating from red seaweed of the algae class *Rhodophyceae*. It is an anionic, linear polymer consisting of 1,3α-1,4β-galactans repeating units with one (κ-), two (ι-) or three (λ-) sulfates per disaccharide unit [157,158]. Three main types of carrageenan can be obtained with a similar chemical structure, namely, kappa (κ), iota (ι) and lambda (λ) [8]. Iota- and kappa-carrageenans self-associate into helical structures in ionic fluid and form flexible or rigid gels, respectively. Kappa-carrageenan hydrogels exhibit thermoreversibility because of their large and flexible spiral molecules, and they perform gelation in conditions without salt and with potassium ions to form the strongest structure [159]. Lambda-carrageenans are non-gelling and do not form helices. Industrially obtained carrageenans are most frequently a combination of a few carrageenans types, with κ- and ι- carrageenans mixtures being the most usual [4]. Carrageenans are used in the food industry [160,161,162] and in some biomedical and pharmaceutical uses such as wound healing [163,164], controlled drug release [165], cartilage scaffold and 3D bioprinting and tissue engineering [166] thanks to their biocompatibility [8].

A total of 1525 patent applications and granted patents were found in the Espacenet database, filtered according to title, abstract and claims for carrageenan in the period from 1968 to 27 May 2023 [13]. The search was narrowed to involve only patents with the keyword “carrageenan” in the title and an earliest priority date after 2013. Carrageenan was the subject of 390 patent applications, of which 172 patents were granted, and some of selected documents are described and summarized in Table 9.

The aim of patent JP6689052B2 is to provide a carrageenan with excellent compatibility with other ingredients in pharmaceutical or food preparations. The divalent cation content of carrageenan can be significantly reduced by carrying out the divalent cation reducing step using potassium citrate or sodium citrate and removing the citrate solution from the mixed carrageenan and citric acid solution at the time of carrageenan production [167]. Using the divalent cation-containing low-content carrageenan obtained by the presented method can eliminate or reduce insoluble salts in pharmaceutical preparations or the food industry.

Patent KR101354180B1relates to a method for producing a butter cake using a butter cream with enhanced cohesiveness of carrageenan, wherein the shape of the cake is not deformed by an external impact or then temperature due to an improved thickening effect and a coagulation force [168].

Patent CN104921964B solves the technical problem of κ-carrageenan having poor solubility, a large molecular weight and easy gelation. Because of this, its application in antioxidant activity is limited [169]. The patent provides a carrageenan-tea polyphenol microsphere with oxidation resistance. The core material of the microsphere is tea polyphenol, and the wall is low-molecular-weight carrageenan (the mass ratio of core material to wall material is 1:1~4). The obtained antioxidant-resistant carrageenan-tea polyphenol microsphere may be used for preparing a sleeping mask.

Granted patent CN104894100B protects a method for preparing immobilized κ-carrageenase [170]. The optimal reaction conditions (free κ-carrageenase and glutaraldehyde concentrations, crosslinking time, fixed time and temperature), storage stability and recyclability of the immobilized κ-carrageenase were studied. The theoretical foundation for the application of immobilized κ-carrageenase was laid. Another object of patent is the preparation of κ-carrageenan oligosaccharide by hydrolyzing a κ-carrageenan substrate using the immobilized κ-carrageenase which was prepared in two steps.

The development of a novel gel based on a mixture of ĭ- and κ- types carrageenan, which is moderately soft, hardy and flexible under conditions of low concentrations of ĭ-type and κ-type carrageenans, was the main subject of patent CN105778122B, which also provides a preparation method by means of swelling and cooling [171]. The ĭ-type carrageenan and κ-type carrageenan mixed gel uses two kinds of carrageenan as a raw material and alanine as a gel-promoting agent to make the gel, and the mixed carrageenan is 0.5–1.0 at a low concentration. The carrageenan mixed gel has high transparency, moderate hardness and elasticity and is not easily dehydrated, thereby overcoming the disadvantages of gel formation of ĭ-type and κ-type carrageenan.

Patent CN106556654B, belonging to the agricultural product quality and safety testing field, relates to a method for kappa-carrageenan detecting in livestock meat, especially t application of a liquid chromatography–tandem mass spectrometry method [172].

Patent CN112430290B discloses a κ-carrageenan-based high-strength double physical crosslinked hydrogel and an obtaining method thereof [173]. The κ-carrageenan-based high-strength double physical crosslinked hydrogel can be obtained via triggering or photo-initiating. The preparation method is simple and easy, the preparation efficiency is high, and the prepared hydrogel has excellent mechanical properties.

The aim of the patent CN112220046B is to provide a compound nutritional fortifier containing selenized carrageenan which can be absorbed, synergized and applied as a supplement with beneficial elements, such as selenium and iron, for improving the human body’s immunity [174]. The macromolecular selenized carrageenan degrades into small fragments of oligosaccharides that are more easily absorbed, improving its bioavailability. Mixing the selenized carrageenan with vitamin E and iron additionally increases safety and avoids overdose.

A carrageenan sulfatase, along with the enzyme, is used to degrade κ-carrageenan CN112522235B [175]. By removing the sulfate group, its content in the κ-carrageenan is reduced and the desulfurization speed is higher, meaning the rheological properties and the gel strength of kappa-carrageenan can be controlled by this enzyme.

Carrageenan is an ancient, natural, plant-based food ingredient. Carrageenan-based hydrogels have great advantages and applications in the food industry, and they are also promising natural polysaccharides for biomedical applications, especially for controlled and sustained drug delivery, tissue engineering and wound healing. The abundance of functional groups is very convenient for further chemical modification for enhancement of the physicochemical properties of these hydrogels, e.g., the hydrophilic surface could be activated by hydrophobic modification. Similar to previously noted approaches, scientists make additional structural modifications for innovative products and processes with the aim of enhancing their applications and ensuring their safe usage based on clinical trials. Many therapeutic applications of carrageenan-based hydrogels are in the experimental stages; we await validation of their effectiveness and objective capability.

#### 2.2.5. Cellulose

Cellulose is the most abundant polysaccharide worldwide. It is produced in nature as a structural polymer in plant life, and it is also formed by some algae, fungi and bacteria. Cellulose is a linear, hydrophobic homopolymer derived from a dimer of glucose as repeating cellobiose units, especially two β-D-glucopyranose units connected by β-(1→4) glycosidic bonds which form a ribbon structure, stabilized by many strong intramolecular hydrogen bonds [8,176]. They form 3D matrix that is responsible for its crystalline form and favorable cellulose tensile features that contribute to the rigidity of plant walls and insolubility in water and common solvents [177]. The enzyme cellulase is able to hydrolyze these bonds and break down cellulose [178].

**Figure 5:**
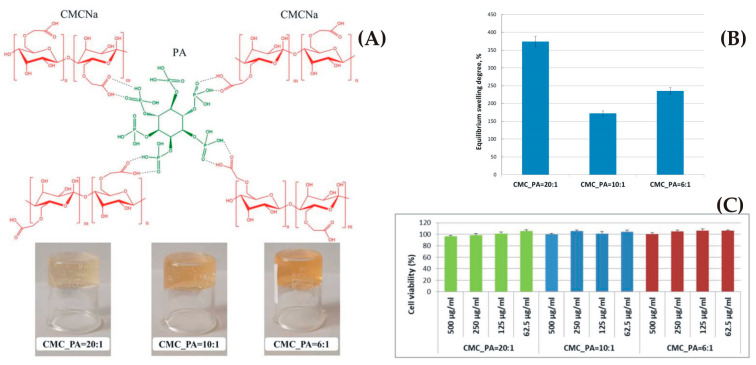
(**A**) Schematic illustration for the formation of CMC/PA hydrogels and the inverted vial test. (**B**) Equilibrium degree of swelling of the hydrogels at different CMC/PA molar ratios. (**C**) Cell viability of normal human dermal fibroblasts exposed to hydrogel extracts (500/250/125/62, 5 µg/mL) for 24 h. Experiments were conducted in triplicate, and treated cell viability was expressed as percentage of control cells’ viability. Graphical data were expressed as means ± standard error of the mean. Reprinted from ref. [187] under open access creative common CC-BY license.

Cellulose, as one of the renewable and easily accessible materials, is extensively used in the food [179], paper [180], textile [181], packaging [182], pharmaceutical [183,184] and biomedical [185] fields. Hydrogels based on cellulose and its derivatives possess structural and morphological improvements, e.g., pore sizes and swelling ratio enhancement, because of the repulsive forces of intramolecular carboxyl groups [186]. For example, new hydrogels based on carboxymethyl cellulose physically crosslinked with phytic acid—an unconventional crosslinking agent (Figure 5)—demonstrated antibacterial properties [187]. Biocompatibility evaluated on fibroblast cells show improved cell viability. Phytic acid, apart from its antioxidant and antibacterial properties, can additionally improve the biological properties, stability and non-toxicity. Procaine, as a model drug, was encapsulated into the hydrogels for in vitro studies and showed drug release dependency on the phytic acid content.

**Figure 6:**
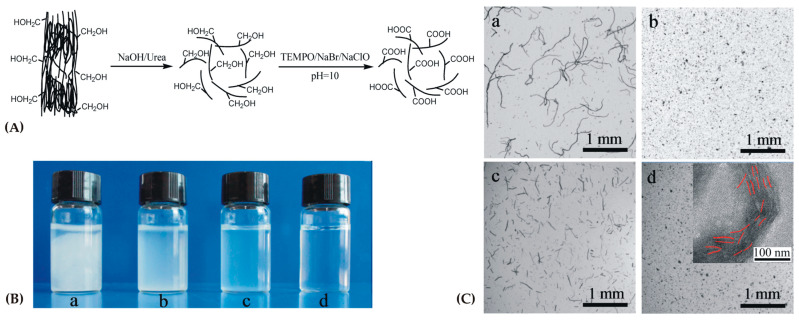
(**A**) Preparation of NaOH/urea-treated TEMPO-oxidized cellulose using a two-step process. (**B**) Dispersion states of (**a**) pristine cellulose; (**b**) NaOH/urea-treated cellulose; (**c**) direct TEMPO-oxidized cellulose; and (**d**) NaOH/urea-treated TEMPO-oxidized cellulose. (**C**) Optical microphotographs of (**a**) pristine cellulose; (**b**) NaOH/urea-treated cellulose; (**c**) direct TEMPO-oxidized cellulose; and (**d**) NaOH/urea-treated TEMPO-oxidized cellulose. (Inset in (**d**) is the TEM image of NaOH/urea-treated TEMPO-oxidized cellulose.) Reprinted from ref. [188] under open access creative common CC-BY license.

Water-soluble 2,2,6,6-tetramethylpiperidine-1-oxyl (TEMPO)-oxidized cellulose was obtained from bamboo-dissolving pulp [188]. In the first step, the cellulose crystals were destroyed using NaOH/urea solution, and the obtained cellulose powder had decreased crystallinity (Figure 6); in the second step, the cellulose powder was oxidized by TEMPO oxidation. The TEMPO-oxidized cellulose with a high degree of oxidation was water soluble for intermediates and polyelectrolytes.

In the worldwide Espacenet database, 8514 patent documents were found, filtered according to title, abstract and claims for biopolymer hydrogels based on cellulose in the period from 1915 to 27 May 2023 [13]. The search was narrowed to involve only patents with the keyword “cellulose” in the title, and an earliest priority date after 2014. Cellulose biopolymer hydrogels were subject of 281 patent application, with registered 143 patents. For the last ten years, 132 patents were granted and chosen patents are analyzed below (Table 10).

Patent US10207252B2 discloses pristine cellulose nanocrystals with functionalized surfaces, incorporated in hydrogel beads applied as adsorbents for pollutants in water treatment [189]. These hydrogel beads were obtained from sodium alginate biopolymer by ionic crosslinking and using calcium chloride. Cellulose nanocrystal is functionalized by polymer grafting, amine or carboxyl functionalization, or amine coating. Antibacterial activity against both Gram-positive (*Bacillus subtilis*) and Gram-negative (*Escherichia coli*) bacteria were tested.

Patent US9650742B2 provides a production method for value-added hemicellulose, extracted from a cellulosic pulp and paper industry byproducts, and for commercial uses [190]. The patent describes a process for converting a hemicaustic extract into a high-viscosity-hydrogel-forming material. This invention provides a hydrogel-forming material that could be used as a thickener in many applications (e.g., in construction, in oil and gas well drilling, in mining, as a thickener in lubricants and paints). Additionally, hemicaustic extract from the paper and pulp industry is converted into a hydrogelling material and is able to produce practically clear fluids after resolving in water.

Patents EP3335695B1 [191] and EP3335696B1 [192] relate to obtaining a method for drying cell-free adipose tissue extracts into a hydrogel based on nanofibrillar cellulose, polyethylene glycol and trehalose. They protect a obtaining method for freeze-dried medical hydrogels consisting of nanofibrillar cellulose, a dried cell-free adipose tissue extract in a freeze-dried biomedical hydrogel and one or more therapeutic agent(s), body fluids or cells, with the option of its delivering. Dried hydrogels comprise nanofibrillar cellulose chosen from anionically or cationically modified nanofibrillar cellulose, unmodified nanofibrillar cellulose and catalytically oxidized nanofibrillar cellulose via the 2,2,6,6-tetramethylpiperidinyl-1-oxy free radical (TEMPO). Surprisingly, it was found that through cryoprotectants’ combination of both trehalose and polyethylene glycol in the freeze-drying process of cell-free tissue extracts in nanofibrillar cellulose hydrogels, it is possible to obtain a dried product that can be redispersed or rehydrated in a form that restores the native features of the tissue extract in its hydrogel; that is, the dried product can be re-gelled.

The nanofibrillar cellulose hydrogel disclosed by patent EP3572434B1 may consist of azido-modified nanofibrillar cellulose with a substituent (the formula for which is O-(CH_2_)_n-_S(O)_m_-L_1_-N_3_, where 1 < n < 10; m is 0 or 1; L_1_ is a linker), which is attached to a carbon atom of glucosyl units (one or more) of azido-modified nanofibrillar cellulose, and which, accordingly, forms an ether bond with carbon atom [193]. This patent protects applications of the mentioned hydrogel or ligand-modified nanofibrillar cellulose hydrogel for the culturing, passaging, maintaining, isolating, transporting, propagating, transplanting or differentiating of tissues or cells.

Patent CN111359007B provides a modified bacterial cellulose hydrogel dressing prepared by 5–10% modified bacterial cellulose, 2–3% toluene isocyanate-activated polyethylene glycol, 0.1–3% active substances (actaprid, acemetacin, ampiroxicam, amfenac, ibuprofen indomethacin, etodolac, ketoprofen, zaltoprofen, diclofenac, sulindac, celecoxib, tiaprofenic acid, tenoxicam, naproxen, piroxicam, felbinac, pranoprofen, flurbiprofen, mefenamic acid, meloxicam, rofecoxib, loxoprofen or lornoxicam) and water [194]. The hydrogel dressing has great water-holding capacity, high crystallinity, is resistant to liquid, gas and electrolytes, has great strength and tension and has good permeability.

The preparation method of an antibacterial wheat straw cellulose composite hydrogel, disclosed in a patent CN112724422B, comprises the following steps: wheat straw pretreatment, potato pretreatment and the preparation of wheat straw cellulose composite hydrogel [195]. The obtained wheat straw cellulose composite hydrogel, used as the raw material to extract cellulose, reduces the waste of resources and is applied in the preparation of a medicine for inhibiting Gram-negative or Gram-positive bacterial infection (against *Escherichia coli* and *Staphylococcus aureus*). The water retention of the composite hydrogel increases with the decrease of potato flour content and keeps moisture up to 48 h at 40 °C, which can provide sufficient nutrients for the soil after dehydration and can also be applied in agriculture.

Patent CN113150319B discloses a high-efficiency self-healing hydrogel which is reinforced by cellulose nanocrystals, together with a hydrazide-modified hydrogel matrix material with a disulfide bond and a crosslinked network structure formed therewith [196]. The obtained high-efficiency self-healing hydrogel has a tensile strength of 5–45 kPa, a water absorption rate of 500–20,000% and a tensile strength recovery rate after self-healing of ≥95%. Used hydrazide-modified hydrogel matrix material with disulfide bond is hyaluronic acid.

Patent CN114230719B provides a double crosslinked cellulose-based hydrogel prepared by cellulose from dried pineapple pomace, with added acrylic acid and 2-acrylamide-2-methylpropanesulfonic acid in the cellulose solution, initiated by cold plasma [197]. The measured tensile strength of the double crosslinked cellulose-based hydrogel was 1.33–2.15 MPa, and for comparison, the tensile strength of the traditional cellulose hydrogel is about 0.16 MPa. The hydrogel is applied to the adsorption of heavy metal ions and can be reused and degraded as a biosorbent. The adsorption time is 45–60 min, and the heavy metal ions removal rate reaches 56–72%, which is 45–90% higher than that of traditional cellulose hydrogel. Using pineapple peel as raw material, the expansion of its application and solving the problem of heavy metal ion pollution in water are conducted at the same time so as to achieve the dual purpose of treating waste with waste.

Cellulose is a low-cost, available, biodegradable natural polysaccharide with very good functional properties and ease of preparation. Its main feature is its swelling ability when in contact with water or aqueous fluids. Cellulose-based hydrogels are extensively applied as biosensors, functional food for delivering nutrients, as active/smart food packaging, as adsorbers for heavy metals, as dyes in wastewater treatment, etc. The chemically crosslinked cellulose-based hydrogels have satisfactory levels of stability and strength. However, the main weakness of the chemical crosslinking method is the toxicity of the residual crosslinkers. Many studies found alternative approaches, e.g., the use of low toxic crosslinkers (such as carbodiimide, borax, sodium trimetaphosphate, *N,N*′-methylene bisacrylamide and polycarboxylic acids or irradiations). Some of the study outcomes include lower crystallinity degree and thermostability. Marking a difference from many analyzed biomaterials, major advancement has been achieved in improving the features of cellulose-based hydrogels in various fields of application. This includes adopted technologies for cellulose-based hydrogels production and the optimized process conditions. New physicochemical approaches to simultaneously control the gelation process and interactions between the obtained hydrogel and native tissues could additionally improve their applications in tissue engineering and drug delivery.

## 3. Concluding Remarks and Perspectives

This review provides a systematized survey of granted patents considering only the improvements and innovations of biopolymer-based hydrogels (from 2010 to 2023) in the latest published papers. A patentability analysis of selected documents based on natural hydrogels based on proteins and polysaccharides is provided.

Nowadays, information is kept secret (i.e., as “know-how) or usually protected by patent rights, especially in industry. Globalization has demanded that universities also adapt to such a trend and open up for business and international cooperation. This includes effective protection, management of research results and cooperation with industry. As a result of the presented patent search, it was noticed that a large number of patents originate from universities, as well as from industry–academic cooperations and foundations (Table 1, Table 2, Table 3, Table 4, Table 5, Table 6, Table 7, Table 8, Table 9 and Table 10). Additionally, it is interesting to note that currently, above 60% of all patent information comes from China. Monitoring the numerous patent documents originating in China is hard, even with advances in proximity search and machine translation engines. Some time ago, this trend gained a new dimension in accelerated examination: numerous national CN patent applications are being fast granted, i.e., within only a few months of the date of filing, which generates new challenges for applicants, examiners, patent searchers and similar [198].

Biopolymer hydrogels are suitable starting materials for biomaterials, which is obvious by their significant scientific and technological benefits. Because of the characteristics of biopolymer-based materials, such as biocompatibility, particular interactions with living tissue, degradability, responsivity and regrowing resources, biopolymers are very useful. Biopolymers´ usability for biomedical applications, especially as carriers for the controlled delivery of drugs, proteins and nucleic acids, cell binding and proliferation, requires particular modifications and the synthetic designing of the desired properties. Major progress in recent times presents the functional groups’ introduction to allowing controlled crosslinking density and elastic moduli to design better mechanical properties. Novel biopolymer hydrogels ensure better bioactivity, enable physical interactions with bioactive molecules and ensure control of the diffusion rates of incorporated active substances, as well as the conformation, properties and functions of obtained biopolymer materials. Therefore, they have shown great potential.

Despite the numerous investigations into new, innovative biopolymer-based hydrogels, their production processes and the development of new, inventive applications, the biological interactions between cells, tissues and biopolymers still remains under-researched. The summarized properties (advantages, applications and weaknesses) of each kind of hydrogel analyzed in this review are presented in Table 11.

The degradation processes of biopolymer-based hydrogels, and the metabolic processes and immunological body responses to biopolymers, as well as their degradation, are hard to predict and are not sufficiently recognized and understood. They are of most importance in biomedicine, with the aim of controlling the delivering time in vivo, the dosage of released drugs and the produced biological effects. Interdisciplinary collaboration for the integration of knowledge from different disciplines—e.g., bioengineering, medicine, pharmacology—is required in order to put the new biopolymer-based hydrogels into clinical application. Considering the many advantages of biopolymer-based hydrogels, it should be expected that their new applications will advance clinical practice.

This review could be useful in future studies of biopolymer-based hydrogels, and it may serve as a starting point from which to plan and design future research in this particular field.

## Figures and Tables

**Table 1:** Selected relevant patents of fibrin-based hydrogels.

Title	Applicant	Priorities/ Publication No	Earliest Priority	Patent No., [Reference]
Fibrin hydrogels comprising plasmonic nanoparticles	Fundación Para La Investigación Biomédica Del Hospital Univ La Paz [ES] Ct De Investigación Biomédica En Red En Bioingeniería Biomat. Y Nanomedicina Ciber Bbn [ES], Univ Zaragoza [ES]	WO2014198989A1	14 June 2013	ES2527800B1 [20]
Encapsulation and cardiac differentiation of hiPSCs in 3D PEG-fibrinogen hydrogels	University of Auburn [US]	US2019284534A1	11 November 2013	US11371021B2 [21]
Fibrin-HA hydrogel carried with G-CSF slow release system, preparation method and purpose thereof	West China Hospital Sichuan University [CN]	CN106581772A	2 December 2016	CN106581772B [22]
Optimized fibrin-polymer composite hydrogel and use thereof	Korea Inst Ceramic Eng and Tech [KR]	KR20190054778A	14 November 2017	KR101991035B [23]
Method for producing a fibrin biopolymer, means for applying said fibrin biopolymer and method for applying said fibrin biopolymer	Kaivo Pesquisa E Desenvolvimento Em Saude Ltda [BR]	WO2018191801A1	18 April 2017	BR102017008027B1 [24]
Bioactive calcium phosphate/fibrin composite injectable bone repair hydrogel	Shanghai 10th Peoples Hospital [CN]	CN110947034A	27 September 2018	CN110947034B [25]

**Table 2:** Selected relevant patents of silk-based hydrogels.

Title	Applicant	Priorities/ Publication No	Earliest Priority	Patent No., [Reference]
Hydrogel composition for treatment of burn comprising silk fibroin	Univ Hallym Iacf [KR]; Cg Bio Co Ltd. [KR] Industry Academic Cooperation Foundation [KR]	KR20120122377	31 October 2012	KR101462485B1 [34]
Silk hydrogel composition and silk hydrogel mask	Amorepacific corp [KR]	WO2014084567A1	5 June 2014	TWI609699B [35]
Carbonic anhydrase immobilized silk hydrogel and conversion or fixation of carbon dioxide using the same	Pohang University of Science and Technology Industry-University Cooperation Foundation [KR]	KR20150045395A	31 March 2015	KR101745369B1 [36]
Modification method of photocrosslinked silk fibroin and preparation method of in situ drug-loaded hydrogel of photocrosslinked silk fibroin applicants	Zhejiang Sci-Tech University [CN]	CN201710136076A	8 March 2017	CN106977670B [37]
Method using mulberry silk to prepare viscose silk fibroin hydrogel	Zhejiang Sci-Tech University [CN]	CN201710677080A	9 August 2017	CN107619481B [38]
Injectable silk fibroin porous hydrogel and preparation method thereof	Jiangxi Silk Biotechnology Co., Ltd. [CN]	CN201910176011A	8 March 2019	CN109851819B [39]
Silk fibroin hydrogel for treatment of intrauterine adhesion	Wenzhou Medical University [CN]	CN201910374312A	24 April 2019	CN110064077B [40]
Silk fibroin conductive hydrogel and preparation method thereof	University of Qingdao [CN]	CN110305339A	5 July 2019	CN110305339B [41]
Anti-adhesion hydrogel-silk stent composite membrane as well as preparation method and application thereof	Zhejiang University [CN]	CN202011046754A	29 September 2020	CN112316219B [42]

**Table 3:** Selected relevant patents of collagen-based hydrogels.

Title	Applicant	Priorities/ Publication No	Earliest Priority	Patent No., [Reference]
Radiation crosslinked collagen gel, and preparation method and usage method thereof	Sewon Cellontech Co., Ltd. [KR]	KR20110005588A WO2012099293A1	19 January 2011	KR101272484B1; EP2666462B1; [52]
Biofabrication techniques for the implementation of intrinsic tissue geometries to an in vitro collagen hydrogel	University of South Carolina; Musc Found for Res Dev [US]	US201462055688P WO2016049625A1	26 September 2014	US10730928B2 [53]
A composite collagen hydrogel material, an implantable ophthalmic device comprising such material and methods of producing the composite collagen hydrogel material and the implantable ophthalmic device	Linkocare Life Sciences AB [SE]	SE1551698A WO2017108794A1	22 December 2015	EP3393534B1 US10568987B2 CN108601861B [54]
Collagen and collagen-like peptide based hydrogels, corneal implants, filler glue and uses thereof	Hyderabad Eye Res Found [US]	WO2018069873A1	13 October 2016	US11426492B2 [55]
Osteoinductive collagen based composite hydrogel and preparation method thereof	University of Huazhong Agricultural [CN]	CN201810280683A	2 April 2018	CN108543115B [56]
Oriented conductive collagen hydrogel, biomimetic conductive nerve scaffold material and preparation method of oriented conductive collagen hydrogel and biomimetic conductive nerve scaffold material	University of Sichuan [CN]	CN201910459198A	29 May 2019	CN110124113B [57]
Method for preparation of succinylated collagen-fibrinogen hydrogel	University Industry Cooperation Group Kyung Hee Univers [KR]	WO2021085818A1	31 October 2019	KR102119693B [58]
Temperature-sensitive collagen-based hydrogel loaded with bioactive polypeptides and preparation method of temperature-sensitive collagen-based hydrogel	University of Fuzhou [CN]	CN202010125956A	27 February 2020	CN111184917B [59]
Collagen for preparing hydrogel and preparation method of collagen	Jiangnan University; Jiangsu Inst Parasitic Diseases [CN]	CN202011491036A	17 December 2020	CN112521491B [60]
Absorbable hydrogel skin repair scaffold constructed on the basis of recombinant human collagen, preparation method and use method thereof	Changzhou Zhonghui Medical Instrument Co., Ltd. [CN]	CN202110042254A	13 January 2021	CN112717200B [61]

**Table 4:** Selected relevant patents of keratin-based hydrogels.

Title	Applicant	Priorities/ Publication No	Earliest Priority	Patent No., [Reference]
Keratin-based hydrogels	University of South Carolina [US]	US201662423454P	1 November 2016	US10723774B2 [72]
Waterborne polyurethane acrylate grafted keratin hydrogel and preparation method thereof	University of Wenzhou [CN]	CN201711075351A	3 November 2017	CN107828031B [73]
Antibacterial keratin-based hydrogel and preparation method thereof	University of Sichuan [CN]	CN201910963635A	11 October 2019	CN110511405B [74]
Composite hydrogel based on zwitterions and keratin and preparation method thereof	University of Nanjing [CN]	CN202010627818A	2 July 2020	CN111825858B [75]
Keratin hydrogel and preparation method thereof, keratin sponge scaffold and preparation method and application thereof	University of Inner Mongolia Technology [CN]	CN202110684913A	21 June 2021	CN113354840B [76]

**Table 5:** Selected relevant patents of gelatin-based hydrogels.

Title	Applicant	Priorities/ Publication No	Earliest Priority	Patent No., [Reference]
Gelatin derivative, crosslinked gelatin hydrogel and porous body thereof, and methods for producing them	Nat Inst Materials Science [JP]	JP2017112053A	6 June 2017	JP7050296B2 [89]
Application of L-3,4-dihydroxyphenylalanine immobilized in the gelatin hydrogel matrices	Politechnika Wroclawska [PL]	PL42298317A	27 September 2017	PL237373B1 [90]
Injectable hydrogel hemostatic based on marine-derived gelatin as well as application and application method of hemostatic	Shenzhen Inst Adv Tech [CN]	CN202010463576A	27 May 2020	CN111632189B [91]
Gelatin-based low-temperature injection anti-inflammatory antibacterial viscous hydrogel as well as preparation method and application thereof	University of Beijing [CN]	CN202110548620A	19 May 2021	CN113230448B [92]
Methyl propenyl gelatin hydrogel grafted with photoinitiator molecules as well as preparation method and application of methyl propenyl gelatin hydrogel	University of Northwestern [CN]	CN202111045848A	7 September 2021	CN113637187B [93]
Fish skin gelatin-based glycolipid hydrogel with double-dynamic crosslinking function as well as preparation method and application of fish skin gelatin-based glycolipid hydrogel	University of Hainan [CN]	CN202210091682A	26 January 2022	CN114213682B [94]

**Table 6:** Selected relevant patents of chitosan-based hydrogels.

Title	Applicant	Priorities/ Publication No	Earliest Priority	Patent No., [Reference]
Shape memory chitosan hydrogel and preparation method thereof	Wuhan University [CN]	CN202110152190A	3 February 2021	CN112940287B [106]
Cell cultivation in chitosan alginate hydrogel beads	Université de Liège [BE]	EP10154712A WO2011104131A1	25 February 2010	EP2538987B1 [107]
Chitosan hydrogel for repairing nerve tissue	Université Claude Bernard Lyon I; Institut National des Sciences Appliquées de Lyon [FR], Univ. Jean Monnet de Saint-Etienne; Centre National de la Recherche Sci.; Inserm Institut Nat. de la Santé Et de la Rech.e Médicale; Univ. Pierre et Marie Curie [FR]	FR1257006A	19 July 2012	EP2874672B1 [108]
Crosslinked chitosan-lactide hydrogels	The Board of Trustees of the Leland Stanford Junior University [US]	US201361810101P WO2014169045A1	9 April 2013	US9814779B2 [109]
The method of obtaining the aqueous solution of chitosan, chitosan composition, chitosan aerosol, the method of producing the chitosan hydrogel membrane and the method of producing chitosan-protein biopolymer mater.	Politechnika Gdanska [PL]	PL2013000085W WO2014014370A3	26 June 2013	EP2920240B1 [110]
Method for the production of hydrogel comprising chitosan and negatively charged polyelectrolytes, and cellular, porous material resulting from said hydrogel	Université de Lille; Centre National de la Recherche Scientifique [FR]; Institut National de la Santé et de la Recherche Médicale (INSERM) [FR]; Centre Hospitalier Régional Universitaire de Lille [FR]	FR1556283A	2 July 2015	EP3317326B1 [111]
Temperature sensitive hydrogel composition including nucleic acid and chitosan	Pharmaresearch Products Co., Ltd. [KR]	KR20160015112A	5 February 2016	EP3412313B1 [112]
Macroporous chitosan-polyacrylamide hydrogel microspheres and preparation thereof	Trustees of Tufts College [US]	US201662353273P	22 June 2016	US11161958B2 [113]
Chitosan hydrogels for accelerating involution and preventing infection of the mammary gland at drying-off	Her Majesty the Queen in Right of Canada as Represented by the Minister of Agriculture and Agri Food [CA]	CA2017050339W	16 March 2017	US10828319B2 [114]
Hydrogel composites comprising chitosan and cellulose nanofibers	Albert-Ludwigs-Universität Freiburg; Université Claude Bernard Lyon 1; Centre National de la Recherche Scientifique—CNRS; Institut Enseignement Supérieur et Recherche en Alimentation Santé Animale Sciences Agronomiques et Environnement (Vet Agro Sup) [FR]	EP18161631A	13 March 2018	EP3765104B1 [115]
Preparation method of high-strength antioxidation chitosan/polydopamine composite hydrogel	South Central University for Nationalities [CN]	CN201910561925A	26 June 2019	CN110237782B [116]
NIR responsive chitosan-based hydrogels for drug delivery system and method for preparing the same	Pukyong National University Industry-University Cooperation Foundation [KR]	KR20200085663A	10 July 2020	KR102372964B1 [117]

**Table 7:** Selected relevant patents of hyaluronic acid-based hydrogels.

Title	Applicant	Publication Number	Earliest Priority	Patent No., [Reference]
Derivates based on hyaluronic acid, capable of forming hydrogels, method of preparation thereof, hydrogels based on said derivatives, method of preparation thereof and use	Contipro Biotech SRO [CZ]	KR20140127286A WO2013127374A1	28 February 2012	KR101953709B1 [128], EP2820051B1
Method for crosslinking hyaluronic acid; method for preparing an injectable hydrogel; hydrogel obtained; use of the obtained hydrogel	Anteis Sa [CH]	FR1301332A WO2014199022A1	11 June 2013	EP3007737B1 [129], US9782490B2
Method for obtaining an injectable hydrogel based on hyaluronic acid containing lidocaine added in powder form, and an alkaline agent, sterilized with heat	Anteis Sa [CN]	FR1359338A WO2015169849A1	27 September 2013	EP3049055B1 [130] US10272181B
Hydrogels of methacrylic hyaluronic acid derivatives for oral enzyme therapy in celiac disease	Nemysis Ltd. [IT]	ITFI20140106A WO2015169849A1	7 May 2014	EP3139961B1 [131]
Microstructure using crosslinked hyaluronic acid hydrogel, and method for producing same	Endo Derma Co., Ltd. [KR]	KR20150022300A WO2016129967A1	13 February 2015	KR101838715B1 [132]
Co-crosslinked hyaluronic acid-silk fibroin hydrogels for improving tissue graft viability and for soft tissue augmentation	Allergan Inc [US]	US201662379045P WO2018039496A1	24 August 2016	US10300169B2 [133]
Hydrogel using, as substrate, hyaluronic acid derivative modified with gallol group and use thereof	Amtixbio Co., Ltd. [KR]	KR20170014855A WO2018143736A1	2 February 2017	KR102099981B1 [134]
Filler having excellent filler properties comprising hyaluronic acid hydrogel	Lg Chemical Ltd. [CN]	KR20180166747A WO2020130684A1	20 December 2018	KR102334794B1 [135]
Filler comprising hyaluronic acid hydrogel having excellent filling properties	Lg Chemical Ltd. [KR]	KR20180167782A WO2020130685A1	21 December 2018	KR102398680B1 [136]
Hydrogel including serotonin-modified hyaluronic acid and use thereof	University of Yonsei Iacf [CN]	CN113574109A WO2020185041A2	14 March 2019	CN113574109B [137]

**Table 8:** Selected relevant patents of alginate-based hydrogels.

Title	Applicant	Publication Number	Earliest Priority	Patent No., [Reference]
Alginate hydrogel fibers and related materials	Mace C.R., Barber J., Laromaine S.A., Whitesides G.M., Cademartiri R. Harvard College [US]	US2013316387A1	12 July 2010	US9090868B2 [147]
Transport of cells in alginate hydrogels	University of Reading [GB]	GB201104711A WO2012127224A1	21 March 2011	EP2688397B1 [148]
Sulfated alginate hydrogels for cell culture and therapy	ETH Zuerich [CH]	EP12007560A WO2014072035A	7 November 2012	EP2916881B1 [149]
FGF-18 formulation in alginate/collagen hydrogels	Ares Trading S.A. A Swiss Company [CH]	EP13199591A WO2015097236A	24 December 2013	EP3086822B1 [150]
Alginate monomer structure with metal crystallite embedded, alginate salt structure with metal crystallite embedded and method of producing alginate hydrogel with metal crystallite incorporated	Hopewang Ent Co., Ltd. [TW]	TW102117077A	14 May 2013	TWI472536B [151] US9499641B2
Alginate hydrogel compositions	Millennium Pharm Inc [US] Takeda Pharmaceuticals Co. [JP]	EP3433282A2	24 March 2016	EP3433282B1 [152]
Method for preparing miniature intelligent calcium alginate hydrogel end operator	Beijing Institute Tech [CN]	CN201910788938A WO2021036200A	26 August 2019	CN110548214B [153]
Hydrogel composition having alginate coupled methacrylate and manufacturing method of hydrogel	Ulsan Nat Inst Science and Tech Unist [KR]	KR20200133490A	15 October 2020	KR102521317B1 [154]
Spraying apparatus and preparing method for hydrogel including a first sprayer and a second sprayer for accommodating a calcium carbonate solution and an alginate solution, respectively	Anti-Microbial Savior Bioteq Co., Ltd. [TW]	TW110104976A	9 February 2021	TWI776389B [155]
Conductive self-healing hydrogel based on sodium alginate, preparation method and application thereof	Ocean University of China [CN]	CN202111271323A	29 October 2021	CN113980294B [156]

**Table 9:** Selected relevant patents of carrageenan-based hydrogels.

Title	Applicant	Publication Number	Earliest Priority	Patent No., [Reference]
Carrageenan having a reduce content of divalent cations and method for producing the same	Teijin Pharma Ltd. [JP]	JP2017066342A	2 October 2015	JP6689052B2 [167]
Manufacturing of butter cake by using carrageenan	CJ Foodville Corp [KR]	KR20130060282A	28 May 2013	KR101354180B1 [168]
Carrageenan-tea polyphenol microsphere with oxidation resistance, as well as preparation method and use thereof	Shanghai Inst Technology [CN]	CN201510310834A	9 June 2015	CN104921964B [169]
Immobilized kappa-carrageenan enzyme and method for preparing kappa-carrageenan oligosaccharide by adopting immobilized kapa-carrageenan enzyme	University of Jimei [CN]	CN201510336185A	17 June 2015	CN104894100B [170]
Iota type carrageenan and Kappa type carrageenan mixed gel and preparation method thereof	University of Dalian Polytechnic [CN]	CN201510863145A	1 December 2015	CN105778122B [171]
Liquid chromatography-tandem mass spectrometry detection method for kappa-carrageenan in livestock meat	Suzhou City Wujiang Distr Agricultural Products Detection Centre Duan Liqin [CN]	CN201610884088A	10 October 2016	CN106556654B [172]
Kappa-carrageenan-based high-strength dual-physical crosslinked hydrogel and preparation method thereof	University of Tianjin [CN]	CN201910792264A	26 August 2019	CN112430290B [173]
Compound nutrition enhancer containing selenized carrageenan and preparation method of compound nutrition enhancer	Qingdao Pengyang Biological Eng Co., Ltd. [CN]	CN112220046A	28 October 2020	CN112220046B [174]
Novel carrageenan sulfatase	Rongcheng Hongpai Marine Biological Tech Co., Ltd. [CN]	CN112522235A	22 December 2020	CN112522235B [175]

**Table 10:** Selected relevant patents of cellulose-based hydrogels.

Title	Applicant	Publication Number	Earliest Priority	Patent No., [Reference]
Pristine and surface functionalized cellulose nanocrystals (cncs) incorporated hydrogel beads and uses thereof	Mohammed Nishil [CA], Grishkewich Nathan [CA], Tam Kam Chiu [CA], Berry Richard	US2016175812A1	22 December 2014	US10207252B2 [189]
Cellulose based hydrogels and process for making the same from hemicaustic byproduct	Rayonier Performance Fibers Llc [US]	US2016168796A1 WO2016094467A1	11 December 2014	US9650742B2 [190]
A method for freeze-drying hydrogel comprising nanofibrillar cellulose, a freeze-dried medical hydrogel comprising nanofibrillar cellulose, and a hydrogel comprising nanofibrillar cellulose	Upm Kymmene Corp [FI]	EP3335695A1 WO2018108341A1	15 December 2016	EP3335695B1 [191] JP6945002B2; US11324701B2
A method for drying cell-free tissue extract in a hydrogel comprising nanofibrillar cellulose and a dried hydrogel comprising nanofibrillar cellulose and cell-free tissue extract	Upm Kymmene Corp [FI], Tampereen Yliopisto [FI], Everfill Oy [FI]	EP3335696A1	15 December 2016	EP3335696B1 [192]
Nanofibrillar cellulose hydrogel	Upm Kymmene Corp [FI]	EP3572434A1 WO2019224314A1	25 May 2018	EP3572434B1 [193] FI129962B
Modified bacterial cellulose hydrogel dressing and preparation method thereof	Reheal Chongqing Biotechnology Co., Ltd. [CN]	CN202010208455A	23 March 2020	CN111359007B [194]
Antibacterial wheat straw cellulose composite hydrogel and preparation method and application thereof	University of Lanzhou [CN]	CN202011591442A	29 December 2020	CN112724422B [195]
Cellulose nanocrystal enhanced efficient self-healing hydrogel and preparation method thereof	University of Wuhan Textile [CN]	CN202110339282A	30 March 2021	CN113150319B [196]
Dual-crosslinked cellulose-based hydrogel prepared from cold plasma and preparation method and application of dual-crosslinked cellulose-based hydrogel	University of South China Science and Tech [CN]	CN202111504454A	10 December 2021	CN114230719B [197]

**Table 11:** Summarized properties of each kind of biopolymer-based hydrogel analyzed.

Hydrogel	Advantages	Weaknesses	Applications
Natural Proteins			
Fibrin	Biodegradable, biocompatible, porous‚ insoluble, elastic, non-globular protein as long fibrous chains	Fast biodegradability, weak mechanical and nanofibrous features, poor printable properties	Surgical glue, sealant and hemostatic agent, promotion bone growth and healing, scaffolds in regenerative medicine, stimulates cell migration, osteoconduction and vascularization, skin, cartilage, cardiac and tissue repair material, composition for tumor destruction, growth factors incorporation, controlled delivery therapeutic agents
Silk fibroin	Excellent biocompatibility, degradability, harmless, non-toxic, stable at physiological conditions, insoluble, non-immunogenic	Fragile texture, low tensile strength, low mechanical properties, potential cytotoxic effects	Tissue engineering, regenerative medicine, controlled drug release, bone regeneration, 3D bioprinting, treating burns and wound healing, corneal repair, molding (without crosslinker), intrauterine implant, anti-adhesion hydrogel-silk scaffold composite film
Collagen	Immense tensile strength, cationic flexible structure, low antigenicity, low immunoreactions, excellent biocompatibility, biodegradability, effortlessly degraded by cells	Fast degradation rate, high shrinkage, weak mechanical strength, opacity	Tissue engineering, implantable ophthalmic devices with incorporated composites, filler glue, corneal implants, osteoinductive hydrogels, thermosensitive collagen-based hydrogel for repairing bone and articular cartilage defects, drugs carrier, scaffold support cells adhesion and proliferation, absorbable skin-repair scaffold, wound healing, chemostactic
Keratin	The toughest natural material, excellent biocompatibility, biodegradability, low cytotoxicity, possibility to mold a definite 3D microstructure.	Hard and difficult processability, denaturation at high temperatures, low commercialization of keratin materials	Porous reproducible ultrafine keratin fibers for controlled cell growth, drug carriers, nerve regeneration, biopolymer absorbents, injectable compositions comprising living cells in tissue regeneration, heavy metal ions absorbent, antibacterial activities, proliferation and infiltration of cells and tissue, formation guided by cells
Gelatin	Excellent biocompatibility, biodegradability, biosafety, non-toxic, adhesive, self-healing, inexpensive, non-immunogenic, soluble in warm aqueous fluids, build gels at low temperatures	Low melting Temperature (30–35 °C), poor mechanical strength, irreversibly hydrolyzed form of collagen	wound repair, cell encapsulation, wound healing, skin substitute, nerve and bone regeneration, reconstruction of soft tissue, 3D bioprinting, colored test for the detection of polyphenol oxidase (tyrosinase), injectable hydrogel hemostatic agent based on marine source gelatin gradually degrade as the wound heals, anti-inflammatory and antibacterial viscous gelatin hydrogel, drug delivery
Polysaccharides			
Chitosan	Cationic natural polymer, biocompatible, bioresorbable, hemocompatible, insoluble in neutral and alkaline fluids, soluble in acidified water (>1% acetic acid) good antimicrobial and antioxidant features, shape-ability onto defect site	Weak mechanical features, mismatching degradation rate, low cell adhesion	Drug delivery, wound dressings, tissue engineering, skin, nerve-cell and bone regeneration, films in food packaging industry, cosmetics, medicine, environmental protection, anti-oxidation composite hydrogel with good mechanical properties, cryogel with improved mechanical properties, excellent water absorption and shape memory, antibacterial activity, cartilage-forming cells, blood vessels embolization, hemostatic, stimulates osteoconduction
Hyaluronic acid	Low immune response, exhibits viscoelasticity depending on the ionic strength and pH value, low immunogenicity, hemostatic, excellent biocompatibility	Weak mechanical features, fast degradation time in living tissue	Regenerative medicine, cosmetology, corneal wound healing, bone and cartilage reparation, drug delivery, 3D bioprinting, scaffolds for articular cartilage or bone tissue treatments, oral treatment of celiac disease, repair skin aging, enlarge human soft tissue (lips, breasts, cheeks, nose, bottom), wrinkle alleviation, support cell or tissue viability or proliferation, prevention of tissue adhesion
Alginate	Cytocompatible, injected as a liquid easily form a hydrogel in situ, inexpensive, self-healing, biodegradable, nontoxicity, immunoprotective, elastic, easy handling, hydrophilic, good shelf life, mechanical properties controlled by divalent cation	Poor dimensional stability, poor tear strength, distortion if unsupported, degradation through ionic exchange with surrounding media, poor biocompatibility	Separation technologies, food industry, dental and orthodontic use, regenerative medicine, tissue engineering, drug delivery, wound healing, desorption of metals, damaged ocular surface cartilage disorders treatment, cartilage injury or osteoarthritis, entrap, encapsulate or transport live cells, a kit for implementation biochemical, diagnostic, cellular, non-cellular analysis, maintaining cell life activities and promoting cell proliferation
Carrageenan	Water-soluble, linear, kappa-carrageenan performs gelation in conditions without salt, with potassium ions forms the strongest structure, thermally, pH, and cation responsive, inexpensive, easy handling, excellent biocompatibility, antioxidant activity	Poor solubility, large molecular weight, easy gelation, but Lambda-carrageenans are non-gelling and do not form helices	Food industry, wound healing, controlled drug release, cartilage scaffold, 3D bioprinting, tissue engineering, skin regeneration, wound healing, antioxidant-resistant carrageenan-tea polyphenol microsphere for the sleeping mask, ĭ-type carrageenan and κ-type carrageenan mixed gel has high transparency, moderate hardness, elasticity, and maintaining hydration, proliferative and chondrogenic potential of encapsulated cells
Cellulose	High mechanical strength, reproducibility, biocompatible, biodegradable, non-toxic, hydrophobic, linear, form a ribbon structure, swell, easy handling, non-irritant, low cytotoxic, crystalline 3D matrix has favorable tensile strength	Toxicity of residual crosslinkers, poor degradability, chemical crosslinking improves solubility and long-term mechanical features	Food, paper, textile, packaging, pharmaceutical industry, antibacterial effects, tissue regeneration, wound dressing and transdermal patches, cartilage tissue engineering biosorbent, hemicaustic extract from the paper and pulp industry, adsorbents for pollutants in water treatment, thickener in many applications (in construction, in oil and gas well drilling, mining, in lubricants and paints)

## Data Availability

Not applicable.

## References

[1] Čolnik M., Knez-Hrnčič M., Škerget M., Knez Ž. (2020). Biodegradable polymers, current trends of research and their applications, a review. Chem. Ind. Chem. Eng. Q..

[2] Saha S., Arshad M., Zubair M., Ullah A., Sharma S., Kumar A. (2019). Keratin as a Biopolymer. Keratin as a Protein Biopolymer.

[3] Neffe A.T., Wischke C., Racheva M. (2013). Progress in biopolymer-based biomaterials and their application in controlled drug delivery. Expert Rev. Med. Devices.

[4] Mahmood A., Patel D., Hickson B., DesRochers J., Hu X. (2022). Recent progress in biopolymer-based hydrogel materials for biomedical applications. Int. J. Mol. Sci..

[5] Fatimi A. (2022). Patentability of Biopolymer-Based Hydrogels. Chem. Proc..

[6] Ilić-Stojanović S., Nikolić L., Nikolić V., Petrović S., Keservani R.K., Sharma A.K., Kesharwani R.K. (2017). Smart hydrogels for pharmaceutical applications. Materials Science and Engineering: Concepts, Methodologies, Tools, and Applications.

[7] Ilić-Stojanović S., Nikolić L., Nikolić V., Ilić D., Ristić I.S., Tačić A., Keservani R.K., Sharma A.K., Kesharwani R.K. (2017). Polymeric matrix systems for drug delivery. Drug Delivery Approaches and Nanosystems.

[8] Fatimi A., Okoro O.V., Podstawczyk D., Siminska-Stanny J., Shavandi A. (2022). Natural Hydrogel-Based Bio-Inks for 3D Bioprinting in Tissue Engineering: A Review. Gels.

[9] Du X., Zhou J., Shi J., Xu B. (2015). Supramolecular hydrogelators and hydrogels: From soft matter to molecular biomaterials. Chem. Rev..

[10] Gharazi S., Zarket B.C., DeMella K.C., Raghavan S.R. (2018). Nature-inspired hydrogels with soft and stiff zones that exhibit a 100-fold difference in elastic modulus. ACS Appl. Mater. Interfaces.

[11] World Intellectual Property Organization. https://www.wipo.int/patents/en/.

[12] European Patent Office Espacenet Patent Search. https://www.epo.org/.

[13] Espacenet Patent Search. https://worldwide.espacenet.com.

[14] Scopus. https://www.scopus.com.

[15] Nehete J., Bhambar R., Narkhede M., Gawali S. (2013). Natural proteins: Sources, isolation, characterization and applications. Pharmacogn. Rev..

[16] (2016). 350th Anniversary of the Discovery of Fibrin (1666–2016) History of Fibrin(ogen). IFRS. Winston-Salem: International Fibrinogen Research Society. https://www.fibrinogen.org/blog/350th-anniversary-of-the-discovery-of-fibrin-1666-2016-history.

[17] Weisel J.W., Litvinov R.I., Parry D., Squire J. (2017). Fibrin formation, structure and properties. Fibrous Proteins: Structures and Mechanisms. Subcellular Biochemistry.

[18] Ahmed T.A.E., Dare E.V., Hincke M. (2008). Fibrin: A versatile scaffold for tissue engineering applications. Tissue Eng. Part B Rev..

[19] Kolehmainen K., Willerth S.M. (2012). Preparation of 3D fibrin scaffolds for stem cell culture applications. JoVE.

[20] Saavedra M., Manuel F., Vilaboa Diaz N., Cebrian Hernando V., Arruebo Gordo M., Jesus S.M., Leyre G.M. (2013). Fibrin Hydrogels Comprising Plasmonic Nanoparticles. ES Patent.

[21] Lipke E.A., Kerscher P., Hodge A.J. (2013). Encapsulation and Cardiac Differentiation of hiPSCs in 3D PEG-Fibrinogen Hydrogels. U.S. Patent.

[22] Xiaojun S., Jian M., Fuxing P. (2016). Fibrin-HA Hydrogel Carried with G-CSF Slow Release System, Preparation Method and Purpose Thereof. CN Patent.

[23] Won Il C. (2017). Optimized Fibrin-Polymer Composite Hydrogel and Use Thereof. KR Patent.

[24] Moacyr R.B., Sartori Barraviera Seabra Ferreira A.S. (2017). Method for Producing a Fibrin Biopolymer, Means for Applying Said Fibrin Biopolymer and Method for Applying Said Fibrin Biopolymer. BR Patent.

[25] Feng C., Yingying J., Zifei Z. (2018). Bioactive Calcium Phosphate/Fibrin Composite Injectable Bone Repair Hydrogel. CN Patent.

[26] Sun W., Gregory D.A., Tomeh M.A., Zhao X. (2021). Silk Fibroin as a Functional Biomaterial for Tissue Engineering. Int. J. Mol. Sci..

[27] Silk. https://www.britannica.com/topic/silk.

[28] Yuan T., Li Z., Zhang Y., Shen K., Zhang X., Xie R., Liu F., Fan W. (2020). Injectable ultrasonication-induced silk fibroin hydrogel for cartilage repair and regeneration. Tissue Eng. Part A.

[29] Kapoor S., Kundu S.C. (2016). Silk protein-based hydrogels: Promising advanced materials for biomedical applications. Acta Biomater..

[30] Mottaghitalab F., Hosseinkhani H., Shokrgozar M.A., Mao C., Yang M., Farokhi M. (2015). Silk as a potential candidate for bone tissue engineering. J. Control. Release.

[31] Rajput M., Mondal P., Yadav P., Chatterjee K. (2022). Light-based 3D bioprinting of bone tissue scaffolds with tunable mechanical properties and architecture from photocurable silk fibroin. Int. J. Biol. Macromol..

[32] Zhang H., Liu Y., Chen C., Cui W., Zhang C., Ye F., Zhao Y. (2020). Responsive drug-delivery microcarriers based on the silk fibroin inverse opal scaffolds for controllable drug release. Appl. Mater. Today.

[33] Wang Q., Zhou S., Wang L., You R., Yan S., Zhang Q., Li M. (2021). Bioactive silk fibroin scaffold with nanoarchitecture for wound healing. Compos. B. Eng..

[34] Park C.H., Lee O.J., Kim J.H., Lee J.M., Ju H.W., Moon B.M., Ryu H.S., Choi W.I., Park H.J., Park C.H. (2012). Hydrogel Composition for Treatment of Burn Comprising Silk Fibroin. KR Patent.

[35] Bae J.H., Choi Y.G., Lee C.K., Jung J.A. (2014). Silk Hydrogel Composition and Silk Hydrogel Mask. TW Patent.

[36] Cha H.J., Kim C., Yang Y.Z., Jo B.H. (2015). Carbonic Anhydrase Immobilized Silk Hydrogel and Conversion or Fixation of Carbon Dioxide Using the Same. KR Patent.

[37] Huang Y., Shao J., Fan Q., Sun G., Meng Y., Xu J. (2017). Modification Method of Photocrosslinked Silk Fibroin and Preparation Method of In-Situ Drug-Loaded Hydrogel of Photocrosslinked Silk Fibroin Applicants. CN Patent.

[38] Yao J., Yuan M., Cai Y., Xu J. (2017). Method Using Mulberry Silk to Prepare Viscose Silk Fibroin Hydrogel. CN Patent.

[39] Tao H., Liu K. (2019). Injectable Silk Fibroin Porous Hydrogel and Preparation Method Thereof. CN Patent.

[40] Zhao Y., Lu C., Yao Q., Xu H., Zheng Y., Lan Q. (2019). Silk Fibroin Hydrogel for Treatment of Intrauterine Adhesion. CN Patent.

[41] Huang X. (2019). Silk Fibroin Conductive Hydrogel and Preparation Method Thereof. CN Patent.

[42] Jin X., Xie T., Hu R., Ni C., Chen D. (2020). Anti-Adhesion Hydrogel-Silk Stent Composite Membrane as well as Preparation Method and Application Thereof.

[43] Gordon M., Hahn R. (2009). Collagens. Cell Tissue Res..

[44] Shoulders M.D., Raines R.T. (2009). Collagen structure and stability. Annl. Rev. Biochem..

[45] Fagerholm P., Lagali N.S., Ong J.A., Merrett K., Jackson W.B., Polarek J.W., Suuronen E.J., Liu Y., Brunette I., Griffith M. (2014). Stable corneal regeneration four years after implantation of a cell-free recombinant human collagen scaffold. Biomaterials.

[46] Farha L.S., Abdulghani M.M. (2017). Clinical and experimental study to evaluate the effect of biphasic calcium phosphate collagen composite (CPCC) on healing of bone defects after oral surgical procedures. AL-Kindy Coll. Med. J..

[47] He Y., Wang J., Si Y., Wang X., Deng H., Sheng Z., Li Y., Liu J., Zhao J. (2021). A novel gene recombinant collagen hemostatic sponge with excellent biocompatibility and hemostatic effect. Int. J. Biol. Macromol..

[48] Lakra R., Kiran M.S., Korrapati P.S. (2022). Collagen scaffold reinforced with furfural for wound healing application. Mater. Lett..

[49] Furtado M., Chen L., Chen Z., Chen A., Cui W. (2022). Development of fish collagen in tissue regeneration and drug delivery. Eng. Regen..

[50] Zhu J., Li Z., Zou Y., Lu G., Ronca A., D’amora U., Liang J., Fan Y., Zhang X., Sun Y. (2022). Advanced application of collagen-based biomaterials in tissue repair and restoration. J. Leather Sci. Eng..

[51] Tang C., Zhou K., Zhu Y., Zhang W., Xie Y., Wang Z., Zhou H., Yang T., Zhang Q., Xu B. (2022). Collagen and its derivatives: From structure and properties to their applications in food industry. Food Hydrocoll..

[52] Yu J.-C., Yeo S.-K., Kim T.-H., Shu D.-S., Chang C.-H. (2011). Radiation Cross-Linked Collagen Gel, and Preparation Method and Usage Method Thereof. KR Patent.

[53] Yost M.J., Rodriguez-Rivera V. (2014). Biofabrication Techniques for the Implementation of Intrinsic Tissue Geometries to an In Vitro Collagen Hydrogel. U.S. Patent.

[54] Rafat M. (2015). A Composite Collagen Hydrogel Material, an Implantable Ophthalmic Device Comprising such Material and Methods of Producing the Composite Collagen Hydrogel Material and the Implantable Ophtalmic Device. EP Patent.

[55] Griffith M., Samanta A., Jangamreddy J.R. (2016). Collagen and Collagen like Peptide Based Hydrogels, Corneal Implants, Filler Glue and Uses Thereof. U.S. Patent.

[56] Hu Y., Yu X., Zhu S., Wu X., Zhao S., Xiong S. (2018). Osteoinductive Collagen Based Composite Hydrogel and Preparation Method Thereof. CN Patent.

[57] Fan H., Wu C., Chen S., Chen L., Sun J., Luo H. (2019). Oriented Conductive Collagen Hydrogel, Biomimetic Conductive Nerve Scaffold Material and Preparation Method of Oriented Conductive Collagen Hydrogel and Biomimetic Conductive Nerve Scaffold Material. CN Patent.

[58] Kwon I.K., Lee J.S., Nah H.R., Heo D.Y., Moon H.J. (2019). Method for Preparing Succinated Collagen-Fibrinogen Hydrogel. KR Patent.

[59] Wang J., Wang S., Huang C., Chen Y., Ding Z., Chen L. (2020). Temperature-Sensitive Collagen-Based Hydrogel Loaded with Bioactive Polypeptides and Preparation Method of Temperature-Sensitive Collagen-Based Hydrogel. CN Patent.

[60] Wang J., Xu F., Cao J. (2020). Collagen for Preparing Hydrogel and Preparation Method of Collagen. CN Patent.

[61] Zhu S., Li H., Lyu H., Qian S. (2021). Absorbable Hydrogel Skin Repair Scaffold Constructed on the Basis of Recombinant Human Collagen, Preparation Method and Use Method Thereof. CN Patent.

[62] Feroz S., Muhammad N., Ratnayake J., Dias G. (2020). Keratin-Based materials for biomedical applications. Bioact. Mater..

[63] Shavandi A., Silva T.H., Bekhit A.A., Bekhit A.E.D.A. (2017). Keratin: Dissolution, extraction and biomedical application. Biomater. Sci..

[64] Donato R.K., Mija A. (2020). Keratin Associations with Synthetic, Biosynthetic and Natural Polymers: An Extensive Review. Polymers.

[65] Smith J.A., Mele E. (2021). Electrospinning and additive manufacturing: Adding three-dimensionality to electrospun scaffolds for tissue engineering. Front. Bioeng. Biotechnol..

[66] Xu H., Cai S., Xu L., Yang Y. (2014). Water-stable three-dimensional ultrafine fibrous scaffolds from keratin for cartilage tissue engineering. Langmuir.

[67] Posati T., Giuri D., Nocchetti M., Sagnella A., Gariboldi M., Ferroni C., Sotgiu G., Varchi G., Zamboni R., Aluigi A. (2018). Keratin-hydrotalcites hybrid films for drug delivery applications. Eur. Polym. J..

[68] Ferroni C., Varchi G. (2021). Keratin-Based Nanoparticles as Drug Delivery Carriers. Appl. Sci..

[69] Carvalho C.R., Costa J.B., Costa L., Silva-Correia J., Moay Z.K., Ng K.W., Reis R.L., Oliveira J.M. (2019). Enhanced performance of chitosan/keratin membranes with potential application in peripheral nerve repair. Biomater. Sci..

[70] Mokrejš P., Huťťa M., Pavlačková J., Egner P. (2017). Preparation of keratin hydrolysate from chicken feathers and its application in cosmetics. JoVE.

[71] Chen H., Gao S., Li Y., Xu H.-J., Li W., Wang J., Zhang Y. (2022). Valorization of Livestock Keratin Waste: Application in Agricultural Fields. Int. J. Environ. Res. Public Health.

[72] Jabbari E. (2016). Keratin-Based Hydrogels. U.S. Patent.

[73] Chai Y., Song Y., Lan Y., Zou X., Chai Y. (2017). Waterborne Polyurethane Acrylate Grafted Keratin Hydrogel and Preparation Method Thereof. CN Patent.

[74] Cheng H., Chen M., Long L., Ren X. (2019). Antibacterial Keratin-Based Hydrogel and Preparation Method Thereof. CN Patent.

[75] Yuan J., Shen J., Wan X., Li P. (2020). Composite Hydrogel Based on Zwitterions and Keratin and Preparation Method Thereof. CN Patent.

[76] Wang K., Shi Z. (2021). Keratin Hydrogel and Preparation Method Thereof, Keratin Sponge Scaffold and Preparation Method and Application Thereof. CN Patent.

[77] Su K., Wang C. (2015). Recent Advances in the Use of Gelatin in Biomedical Research. Biotechnol. Lett..

[78] Chen Y.-J., Cheng H.-W., Yen W.-Y., Tsai J.-H., Yeh C.-Y., Chen C.-J., Liu J.T., Chen S.-Y., Chang S.-J. (2022). The Treatment of Keloid Scars Via Modulating Heterogeneous Gelatin-Structured Composite Microneedles to Control Transdermal Dual-Drug Release. Polymers.

[79] Lu Y., Luo Q., Chu Y., Tao N., Deng S., Wang L., Li L. (2022). Application of gelatin in food packaging: A review. Polymers.

[80] Hanani Z.N., Roos Y.H., Kerry J.P. (2014). Use and application of gelatin as potential biodegradable packaging materials for food products. Int. J. Biol. Macromol..

[81] Shevchenko R.V., Eeman M., Rowshanravan B., Allan I.U., Savina I.N., Illsley M., Salmon M., James S.L., Mikhalovsky S.V. (2014). The in vitro characterization of a gelatin scaffold, prepared by cryogelation and assessed in vivo as a dermal replacement in wound repair. Acta Biomater..

[82] Matheus H.R., Hadad H., Monteiro J.L.G.C., Takusagawa T., Zhang F., Ye Q., He Y., Rosales I.A., Jounaidi Y., Randolph M.A. (2023). Photo-crosslinked GelMA loaded with dental pulp stem cells and VEGF to repair critical-sized soft tissue defects in rats. J. Stomatol. Oral Maxillofac. Surg..

[83] Baghersad S., Bahrami S.H., Mohammadi M.R., Mojtahedi M.R.M., Milan P.B. (2018). Development of biodegradable electrospun gelatin/aloe-vera/poly (ε-caprolactone) hybrid nanofibrous scaffold for application as skin substitutes. Mater. Sci. Eng. C.

[84] Li J., Gao F., Ma S., Zhang Y., Zhang J., Guan F., Yao M. (2020). Control the fate of human umbilical cord mesenchymal stem cells with dual-enzymatically cross-linked gelatin hydrogels for potential applications in nerve regeneration. J. Tissue Eng. Regen. Med..

[85] Nie K., Han S., Yang J., Sun Q., Wang X., Li X., Li Q. (2020). Enzyme-crosslinked electrospun fibrous gelatin hydrogel for potential soft tissue engineering. Polymers.

[86] Liang R., Gu Y., Wu Y., Bunpetch V., Zhang S. (2020). Lithography-based 3D bioprinting and bioinks for bone repair and regeneration. ACS Biomater. Sci. Eng..

[87] Ko Y.G., Kwon O.H. (2020). Reinforced gelatin-methacrylate hydrogels containing poly(lactic-co-glycolic acid) nanofiber fragments for 3D bioprinting. J. Ind. Eng. Chem..

[88] Rao K.M., Kim H.J., Won S., Choi S.M., Han S.S. (2023). Effect of Grape Seed Extract on Gelatin-Based Edible 3D-Hydrogels for Cultured Meat Application. Gels.

[89] Chin K., Kawazoe N., Li X. (2017). Gelatin Derivative, Crosslinked Gelatin Hydrogel and Porous Body Thereof, and Methods for Producing Them. JP Patent.

[90] Labus K., Krystek K. (2017). Application of L-3,4-dihydroxyphenylalanine Immobilized in the Gelatin Hydrogel Matrices. PL Patent.

[91] Zhao X., Bian S., Pan H. (2020). Injectable Hydrogel Hemostatic Based on Marine-Derived Gelatin as well as Application and Application Method of Hemostatic. CN Patent.

[92] Huang J., Tian Z., Su X., Xie W. (2021). Gelatin-Based Low-Temperature Injection Anti-Inflammatory Antibacterial Viscous Hydrogel as well as Preparation Method and Application Thereof. CN Patent.

[93] Xue W., Wang E., Pan J., He Z., Fu C., Wen H., Huang S. (2021). Methyl Propenyl Gelatin Hydrogel Grafted with Photoinitiator Molecules as well as Preparation Method and Application Of Methyl Propenyl Gelatin Hydrogel. CN Patent.

[94] Xia G., Lu Y., Shin H.I., Zhao M. (2022). Fish Skin Gelatin-Based Glycolipid Hydrogel with Double-Dynamic Crosslinking Function as well as Preparation Method and Application of Fish Skin Gelatin-Based Glycolipid Hydrogel. CN Patent.

[95] Mohammed A.S.A., Naveed M., Jost N. (2021). Polysaccharides; classification, chemical properties, and future perspective applications in fields of pharmacology and biological medicine (A review of current applications and upcoming potentialities). J. Polym. Environ..

[96] Tolstoguzov V.B. (2017). Protein-Polysaccharide Interactions. Food Proteins and Their Applications.

[97] Ruocco N., Costantini S., Guariniello S., Costantini M. (2016). Polysaccharides from the marine environment with pharmacological, cosmeceutical and nutraceutical potential. Molecules.

[98] Roehm K.D., Madihally S.V. (2017). Bioprinted chitosan-gelatin thermosensitive hydrogels using an inexpensive 3D printer. Biofabrication.

[99] Bai X., Bao Z., Bi S., Li Y., Yu X., Hu S., Tian M., Zhang X., Cheng X., Chen X. (2018). Chitosan-based thermo/pH double sensitive hydrogel for controlled drug delivery. Macromol. Biosci..

[100] Alven S., Aderibigbe B.A. (2020). Chitosan and Cellulose-Based Hydrogels for Wound Management. Int. J. Mol. Sci..

[101] Garcia C.E.G., Lardy B., Bossard F., Martínez F.A.S., Rinaudo M. (2021). Chitosan based biomaterials for cartilage tissue engineering: Chondrocyte adhesion and proliferation. FHFH.

[102] Yan T., Kong S., Ouyang Q., Li C., Hou T., Chen Y., Li S. (2020). Chitosan-gentamicin conjugate hydrogel promoting skin scald repair. Mar. Drugs.

[103] Shavandi A., Bekhit A.E.-D.A., Sun Z., Ali M.A. (2016). Injectable gel from squid pen chitosan for bone tissue engineering applications. J. Sol-Gel Sci. Technol..

[104] Tian B., Wang J., Liu Q., Liu Y., Chen D. (2021). Formation chitosan-based hydrogel film containing silicon for hops β-acids release as potential food packaging material. Int. J. Biol. Macromol..

[105] Zhang H., Feng M., Fang Y., Wu Y., Liu Y., Zhao Y., Xu J. (2022). Recent advancements in encapsulation of chitosan-based enzymes and their applications in food industry. Crit. Rev. Food Sci. Nutr..

[106] Xiaowen S., Luhe Q., Hongbing D., Yumin D. (2021). Shape Memory Chitosan Hydrogel and Preparation Method Thereof. CN Patent.

[107] Henrotin Y., Kesteloot F., Sanchez C. (2010). Cell Cultivation in Chitosan Alginate Hydrogel Beads. ES Patent.

[108] Nothias F., Soares S., David L., Montembault A. (2012). Chitosan Hydrogel for Repairing Nerve Tissue. EP Patent.

[109] Kim S., Yang Y.P., Yunzhi P. (2013). Crosslinked Chitosan-Lactide Hydrogels. U.S. Patent.

[110] Gorczyca G., Tylingo R., Szweda P., Milewski S., Sadowska M., Zalewska M. (2013). The Method of Obtaining the Aqueous Solution of Chitosan, Chitosan Composition, Chitosan Aerosol, the Method of Producing the Chitosan Hydrogel Membrane and the Method of Producing Chitosan-Protein Biopolymer Material. ES Patent.

[111] Blanchemain N., Martel B., Flores C., Cazaux F., Chai F., Tabary N., Lopez H.M. (2015). Method for the Production of Hydrogel Comprising Chitosan and Negatively Charged Polyelectrolytes, and Cellular, Porous Material Resulting from Said Hydrogel. EP Patent.

[112] Kim I.S., Kim H.G., Hong C.A., Lee S.Y. (2016). Temperature Sensitive Hydrogel Composition Including Nucleic Acid and Chitosan. EP Patent.

[113] Yi H., Jung S., Abel J.H. (2016). Macroporous Chitosan-Polyacrylamide Hydrogel Microspheres and Preparation Thereof. U.S. Patent.

[114] Lacasse P., Lanctôt S., Fustier P., Bégin A., Taherian A.R., Bisakowski B. (2017). Chitosan Hydrogels for Accelerating Involution and Preventing Infection of the Mammary Gland at Drying-Off. U.S. Patent.

[115] Osorio Madrazo A., David L., Montembault A., Viguier E., Cachon T. (2018). Hydrogel Composites Comprising Chitosan and Cellulose Nanofibers. EP Patent.

[116] Zhang A., Jiang Z. (2019). Preparation Method of High-Strength Antioxidation Chitosan/Polydopamine Composite Hydrogel. CN Patent.

[117] Lim K.T. (2020). NIR Responsive Chitosan-Based Hydrogels for Drug Delivery System and Method for Preparing the Same. KR Patent.

[118] Bencherif S.A., Srinivasan A., Horkay F., Hollinger J.O., Matyjaszewski K., Washburn N.R. (2008). Influence of the degree of methacrylation on hyaluronic acid hydrogels properties. Biomaterials.

[119] Buckley C., Murphy E.J., Montgomery T.R., Major I. (2022). Hyaluronic acid: A review of the drug delivery capabilities of this naturally occurring polysaccharide. Polymers.

[120] Kobayashi Y., Okamoto A., Nishinari K. (1994). Viscoelasticity of hyaluronic acid with different molecular weights. Biorheology.

[121] An C., Li H., Zhao Y., Zhang S., Zhao Y., Zhang Y., Yang J., Zhang L., Ren C., Zhang Y. (2023). Hyaluronic acid-based multifunctional carriers for applications in regenerative medicine: A review. Int. J. Biol. Macromol..

[122] Flégeau K., Jing J., Brusini R., Gallet M., Moreno C., Walker L., Bourdon F., Faivre J. (2023). Multidose hyaluronidase administration as an optimal procedure to degrade resilient hyaluronic acid soft tissue fillers. Molecules.

[123] Ratajczak P., Maciejak O., Kopciuch D., Paczkowska A., Zaprutko T., Kus K. (2023). Directions of hyaluronic acid application in cosmetology. J. Cosmet. Dermatol..

[124] Pilloni A., Marini L., Gagliano N., Canciani E., Dellavia C., Cornaghi L.B., Costa E., Rojas M.A. (2022). Clinical, histological, immunohistochemical and biomolecular analysis of hyaluronic acid in early wound healing of human gingival tissues: A randomized, split-mouth trial. J. Periodontol..

[125] Zheng Z., Patel M., Patel R. (2022). Hyaluronic acid-based materials for bone regeneration: A review. React. Funct. Polym..

[126] Jin Y., Koh R.H., Kim S.-H., Kim K.M., Park G.K., Hwang N.S. (2020). Injectable anti-inflammatory hyaluronic acid hydrogel for osteoarthritic cartilage repair. Mater. Sci. Eng. C.

[127] Poldervaart M.T., Goversen B., De Ruijter M., Abbadessa A., Melchels F.P., Öner F.C., Dhert W.J., Vermonden T., Alblas J. (2017). 3D bioprinting of methacrylated hyaluronic acid (MeHA) hydrogel with intrinsic osteogenicity. PLoS ONE.

[128] Wolfova L., Pravda M., Foglarova M., Nemcova M., Niedoba K., Velebny V. (2012). Derivates Based on Hyaluronic Acid, Capable of Forming Hydrogels, Method of Preparation Thereof, Hydrogels Based on Said Derivatives, Method of Preparation Thereof and Use. KR Patent.

[129] Tauzin B.V. (2013). Method for Crosslinking Hyaluronic Acid; Method for Preparing an Injectable Hydrogel; Hydrogel Obtained; Use of the Obtained Hydrogel. EP Patent.

[130] Gavard Molliard S. (2013). Method for Obtaining an Injectable Hydrogel Based on Hyaluronic Acid Containing Lidocaine Added in Powder Form, and an Alkaline Agent, Sterilized with Heat. EP Patent.

[131] Pitarresi G., Palumbo F.S., Giammona G. (2014). Hydrogels of Methacrylic Hyaluronic Acid Derivatives for Oral Enzyme Therapy in Celiac Disease. EP Patent.

[132] Kim J.S., Kwon S.C., Park S.J. (2016). Microstructure Using Cross-Linked Hyaluronic Acid Hydrogel, and Method for Producing Same. KR Patent.

[133] Yu X., Messina D.J., Pavlovic E., Cui C., Smither K.M. (2016). Co-Crosslinked Hyaluronic Acid-Silk Fibroin Hydrogels for Improving Tissue Graft Viability and for Soft Tissue Augmentation. U.S. Patent.

[134] Cho S.W., Lee J.-S., Cho J.H., Lee J.-S. (2017). Hydrogel Using, as Substrate, Hyaluronic Acid Derivative Modified with Gallol Group and Use Thereof. KR Patent.

[135] Jang C., Lee C., Kim J.S., Jung H.T., Lee C.H., So J., Ree H. (2018). Filler Having Excellent Filler Properties Comprising Hyaluronic Acid Hydrogel. KR Patent.

[136] Jang C., Kim Y., Lee H., Lee Myunghan K., Ji S., Jung H.T., So J., Lee C.H., Ree H. (2018). Filler Comprising Hyaluronic Acid Hydrogel Having Excellent Filling Properties. KR Patent.

[137] Zhao S.Y., An X.H. (2019). Hydrogel Including Serotonin-Modified Hyaluronic Acid and Use Thereof. CN Patent.

[138] Pereira L., Cotas J., Pereira L. (2020). Introductory Chapter: Alginates—A General Overview. Alginates—Recent Uses of This Natural Polymer.

[139] Rowley J.A., Madlambayan G., Mooney D.J. (1999). Alginate hydrogels as synthetic extracellular matrix materials. Biomaterials.

[140] Lee K.Y., Mooney D.J. (2012). Alginate: Properties and biomedical applications. Prog. Polym. Sci..

[141] Alginic acid Medium Viscosity 9005-38-3. https://www.sigmaaldrich.com.

[142] Xiang H., Sun-Waterhousea D., Waterhousea G., Cuia C., Ruana Z. (2019). Fermentation-enabled wellness foods: A fresh perspective. Food Sci. Hum. Wellness.

[143] Gheorghita Puscaselu R., Lobiuc A., Dimian M., Covasa M. (2020). Alginate: From Food Industry to Biomedical Applications and Management of Metabolic Disorders. Polymers.

[144] Wei Q., Zhou J., An Y., Li M., Zhang J., Yang S. (2023). Modification, 3D printing process and application of sodium alginate based hydrogels in soft tissue engineering: A review. Int. J. Biol. Macromol..

[145] Kurakula M., Rao G.K., Kiran V., Hasnain M.S., Nayak A.K. (2020). Alginate-based hydrogel systems for drug releasing in wound healing. Alginates in Drug Delivery.

[146] Salih K.A.M., Zhou K., Hamza M.F., Mira H., Wei Y., Ning S., Guibal E., Salem W.M. (2023). Phosphonation of Alginate–Polyethyleneimine Beads for the Enhanced Removal of Cs(I) and Sr(II) from Aqueous Solutions. Gels.

[147] Mace C.R., Barber J., Sagué A.L., Whitesides G.M., Cademartiri R. (2010). Alginate Hydrogel Fibers and Related Materials. U.S. Patent.

[148] Connon C.J., Cave R.A., Khutoryanskiy V., Wright B. (2011). Transport of Cells in Alginate Hydrogels. EP Patent.

[149] Zenobi-Wong M., Palazzolo G., Mhanna R., Becher J., Möller S., Schnabelrauch M. (2012). Sulfated Alginate Hydrogels for Cell Culture and Therapy. EP Patent.

[150] Canal F., Lo Presti C. (2013). FGF-18 Formulation in Alginate/Collagen Hydrogels. EP Patent.

[151] Yung-Pin L. (2013). Alginate Monomer Structure with Metal Crystallite Embedded, Alginate Salt Structure with Metal Crystallite Embedded and Method of Producing Alginate Hydrogel with Metal Crystallite Incorporated. TW Patent.

[152] Garigapati V.R., Hoshino T., Garle A. (2016). Alginate Hydrogel Compositions. EP Patent.

[153] Wang H., Shi Q., Zheng Z., Liu S., Tan J., Huang Q., Fukuda T. (2019). Method for Preparing Miniature Intelligent Calcium Alginate Hydrogel End Operator. CN Patent.

[154] Cha C.N., Choi C.L., Kim S.T. (2020). Hydrogel Composition Having Alginate Coupled Methacrylate and Manufacturing Method of Hydrogel. KR Patent.

[155] Cai Y.-R., Ye Y.-T., Hong Y.-L., Guo J.-Y., Chen Z.-H. Spraying Apparatus and Preparing Method for Hydrogel Including a First Sprayer and a Second Sprayer for Accommodating a Calcium Carbonate Solution and an Alginate Solution Respectively. TW Patent.

[156] Cai C., Liu D., Li J., Yu G., Liu C. (2021). Conductive Self-Healing Hydrogel Based on Sodium Alginate, Preparation Method and Application Thereof. CN Patent.

[157] Carrageenan. https://www.sigmaaldrich.com/RS/en/product/sigma/c1013.

[158] Martins J.T., Cerqueira M.A., Bourbon A.I., Pinheiro A.C., Souza B.W.S., Vicente A.A. (2012). Synergistic effects between κ-carrageenan and locust bean gum on physicochemical properties of edible films made thereof. Food Hydrocoll..

[159] Sankalia M.G., Mashru R.C., Sankalia J.M., Sutariya V.B. (2006). Stability improvement of alpha-amylase entrapped in kappacarrageenan beads: Physicochemical characterization and optimization using composite index. Int. J. Pharm..

[160] Anggraini J., Lo D. (2023). Health impact of carrageenan and its application in food industry: A review. IOP Conference Series: Earth and Environmental Science.

[161] Jin Y., Lu Z. (2023). Preparation of carrageenan/konjac glucomannan/graphene oxide nanocomposite films with high mechanical and antistatic properties for food packaging. Polym. Bull..

[162] Jiang C., Liu T., Wang S., Zou Y., Cao J., Wang C., Hang C., Jin L. (2023). Antioxidant and ammonia-sensitive films based on starch, κ-carrageenan and Oxalis triangularis extract as visual indicator of beef meat spoilage. Int. J. Biol. Macromol..

[163] Sathuvan M., Thangam R., Cheong K.L., Kang H., Liu Y. (2023). κ-Carrageenan-essential oil loaded composite biomaterial film facilitates mechanosensing and tissue regenerative wound healing. Int. J. Biol. Macromol..

[164] Khodaei T., Nourmohammadi J., Ghaee A., Khodaii Z. (2023). An antibacterial and self-healing hydrogel from aldehyde-carrageenan for wound healing applications. Carbohydr. Polym..

[165] Mahdavinia G.R., Hoseinzadeh H., Labib P., Jabbari P., Mohebbi A., Barzeger S., Jafari H. (2023). (Magnetic laponite/κ-carrageenan)@ chitosan core–shell carrier for pH-sensitive release of doxorubicin. Polym. Bull..

[166] Fogde A., Qudsia S., Le T.A., Sandberg T., Huynh T.P. (2021). (Calcium-Phosphate)/Carrageenan Gardens Grown from the Gel/Liquid Interface. Chem. Systems Chem..

[167] Miyuki K., Hideyuki K., Noriko I., Osamu K. (2015). Carrageenan Having a Reduce Content of Divalent Cations and Method for Producing the Same. JP Patent.

[168] Kim G.H., Park G.S., Kim S.Y., Moon D.K. (2013). Manufacturing of Butter Cake by Using Carrageenan. KR Patent.

[169] Feng T., Gao L., Liu M., Bing F., Liu Y., Li M., Xie K., Sang M., Liu T., Wang S. (2015). Carrageenan-Tea Polyphenol Microsphere with Oxidation Resistance, as well as Preparation Method and Use Thereof. CN Patent.

[170] Xiao A., Cai H., Ni H., Xu C., Zhu Y., Yang Q., Du X., Chen Y. (2015). Immobilized Kappa-Carrageenan Enzyme and Method for Preparing Kappa-Carrageenan Oligosaccharide by Adopting Immobilized Kappa-Carrageenan Enzyme. CN Patent.

[171] Wen C., Gao X., Guo Y., Cong S., Song S., Qi H., Chen X., Zhang Q. (2015). Iota Type Carrageenan and Kappa Type Carrageenan Mixed Gel and Preparation Method Thereof. CN Patent.

[172] Duan L., Tao L., Wang J., Liu M., Chen F., Yang G., Zhuang L. (2016). Liquid Chromatography-Tandem Mass Spectrometry Detection Method for Kappa-Carrageenan in Livestock Meat. CN Patent.

[173] Liu X., Zhao L., Zheng Q., Liu Y., Wang S., Wang J. (2019). Kappa-Carrageenan-Based High-Strength Dual-Physical Cross-Linked Hydrogel and Preparation Method Thereof. CN Patent.

[174] Yang Y., Zhang Y., Wei L. (2020). Compound Nutrition Enhancer Containing Selenized Carrageenan and Preparation Method of Compound Nutrition Enhancer. CN Patent.

[175] Zhang D., Xu J., Zhang D., Song Y., Xu F. (2020). Novel Carrageenan Sulfatase. CN Patent.

[176] Lourenço A., Araújo S., Gominho J., Evtuguin D. (2020). Cellulose Structural Changes during Mild Torrefaction of *Eucalyptus* Wood. Polymers.

[177] Okoro O.V., Amenaghawon A., Podstawczyk D., Alimoradi H., Khalili M.R., Anwar M., Milan P.B., Nie L., Shavandi A. (2021). Fruit pomace-lignin as a sustainable biopolymer for biomedical applications. J. Clean. Prod..

[178] Bayer E., Chanzy H., Lamed R., Shoham Y. (1998). Cellulose, cellulases and cellulosomes. Curr. Opin. Struct. Biol..

[179] Ahmad Khorairi A.N.S., Sofian-Seng N.S., Othaman R., Abdul Rahman H., Mohd Razali N.S., Lim S.J., Wan Mustapha W.A. (2023). A review on agro-industrial waste as cellulose and nanocellulose source and their potentials in food applications. Food Rev. Int..

[180] Hwang Y., Kang K.Y., Yang B.S., Potthast A., Jeong M.J. (2023). Minimally invasive evaluation of cellulose paper degradation using water-soluble carbohydrates. Cellulose.

[181] Stanescu M.D. (2023). Applications of enzymes in processing cellulosic textiles—A review of the latest developments. Cellul. Chem. Technol..

[182] Kumar A., Ramakanth D., Akhila K., Gaikwad K.K. (2023). Influence of halloysite nanotubes/microfibrillated cellulose on pine leaves waste based ethylene scavenging composite paper for food packaging applications. Appl. Clay Sci..

[183] Yang M., Abdalkarim S.Y.H., Yu H.Y., Asad R.A., Ge D., Zhou Y. (2023). Thermo-sensitive composite microspheres incorporating cellulose nanocrystals for regulated drug release kinetics. Carbohydr. Polym..

[184] Kumari P., Seth R., Meena A., Sharma D. (2023). Enzymatic synthesis of cellulose nanocrystals from lemongrass and its application in improving anti-cancer drug release, uptake and efficacy. Ind. Crops Prod..

[185] Fuster M.G., Moulefera I., Muñoz M.N., Montalbán M.G., Víllora G. (2023). Synthesis of Cellulose Nanoparticles from Ionic Liquid Solutions for Biomedical Applications. Polymers.

[186] Kabir S.F., Sikdar P.P., Haque B., Bhuiyan M.R., Ali A., Islam M. (2018). Cellulose-based hydrogel materials: Chemistry, properties and their prospective applications. Prog. Biomater..

[187] Ghilan A., Nita L.E., Pamfil D., Simionescu N., Tudorachi N., Rusu D., Rusu A.G., Bercea M., Rosca I., Ciolacu D.E. (2022). One-Step Preparation of Carboxymethyl Cellulose—Phytic Acid Hydrogels with Potential for Biomedical Applications. Gels.

[188] Tang Z., Li W., Lin X., Xiao H., Miao Q., Huang L., Chen L., Wu H. (2017). TEMPO-oxidized cellulose with high degree of oxidation. Polymers.

[189] Mohammed N., Grishkewich N., Tam K.C., Berry R. (2014). Pristine and Surface Functionalized Cellulose Nanocrystals (Cncs) Incorporated Hydrogel Beads and Uses Thereof. U.S. Patent.

[190] Hamed O.A., Krzywanski R.S. (2014). Cellulose Based Hydrogels and Process for Making the Same from Hemicaustic Byproduct. U.S. Patent.

[191] Auvinen V.-V., Paukkonen H., Yliperttula M., Urtti A., Hakkarainen T., Laurén P., Kunnari M., Laaksonen T., Li M., Luukko K. (2016). A Method for Freeze-Drying Hydrogel Comprising Nanofibrillar Cellulose, a Freeze-Dried Medical Hydrogel Comprising Nanofibrillar Cellulose, and a Hydrogel Comprising Nanofibrillar Cellulose. EP Patent.

[192] Luukko K., Ylikomi T., Sarkanen J.-R. (2016). A Method for Drying Cell-Free Tissue Extract in a Hydrogel Comprising Nanofibrillar Cellulose and a Dried Hydrogel Comprising Nanofibrillar Cellulose and Cell-Free Tissue Extract. EP Patent.

[193] Nuopponen M., Paasonen L., Satomaa T., Aitio O., Helin J. (2018). Nanofibrillar Cellulose Hydrogel. EP Patent.

[194] Feng W., Li Y. (2020). Modified Bacterial Cellulose Hydrogel Dressing and Preparation Method Thereof. CN Patent.

[195] Zhang G., Li Q., Yuan L., Wang L., Lu H. (2020). Antibacterial Wheat Straw Cellulose Composite Hydrogel and Preparation Method and Application Thereof. CN Patent.

[196] Zhou Y., Li S., Wan T., Fan P., Yang H., Gu S., Ye D., Tao Y., Xu W. (2021). Cellulose Nanocrystal Enhanced Efficient Self-Healing Hydrogel and Preparation Method Thereof. CN Patent.

[197] Cheng J., Han Z., Sun D., Ma J. (2021). Dual-Crosslinked Cellulose-Based Hydrogel Prepared from Cold Plasma and Preparation Method and Application of Dual-Crosslinked Cellulose-Based Hydrogel. CN Patent.

[198] Search-Matters Workshop 3: The Chinese IP High-Speed Train: “Accelerated Examination” at CNIPA. https://www.epo.org/news-events/events/conferences/search-matters/programme.html.

